# Regulatory T cell therapies: biological foundations, engineering strategies, and clinical translation

**DOI:** 10.3389/fimmu.2026.1797186

**Published:** 2026-04-17

**Authors:** Charleen Plaisse, Séverine Bézie, Carole Guillonneau

**Affiliations:** Nantes Université, CHU Nantes, Institut national de la santé et de la recherche médicale (INSERM), Center for Research in Transplantation and Translational Immunology, UMR 1064, ITUN, Nantes, France

**Keywords:** allogeneic immunotherapy, CD4+ and CD8+ T cells, cell engineering, cell therapy, immune tolerance, induced pluripotent stem cells, regulatory T cells

## Abstract

Regulatory T cell (Treg) therapy has emerged as a promising strategy to control pathological immune responses in autoimmunity, graft-versus-host disease, and solid organ transplantation. Most clinical studies to date have relied on autologous Tregs expanded ex vivo, an approach that has demonstrated safety and feasibility but remains limited by variable cell quality, restricted scalability, and complex manufacturing requirements. To address these constraints, multiple alternative strategies are being developed, including the induction of regulatory phenotypes in conventional T cells, the engineering of antigen-specific Tregs, and the generation of allogeneic “off-the-shelf” regulatory cell products. In parallel, induced pluripotent stem cells (iPSCs) offer a renewable and standardized source for regulatory T cell generation, enabling extensive genetic engineering and batch consistency. Early-phase clinical trials with CD4^+^ Tregs have established an excellent safety profile, and initial clinical evaluation of CD8^+^ Tregs is now underway. This review provides a comprehensive and comparative analysis of the biological principles, engineering strategies, and translational challenges that shape the development of regulatory T cell-based immunotherapies.

## 1 Introduction

Tregs are central mediators of immune tolerance, maintaining immune homeostasis and preventing pathological immune responses. Initially characterized by the expression of CD4^+^, CD25^high^, CD127^low^, and the lineage-defining transcription factor FOXP3, Tregs suppress autoreactive and alloreactive immune responses, limit inflammation, and protect tissues from immune-mediated damage. The identification of FOXP3 mutations as the cause of severe autoimmune syndromes in both mice and humans established the critical role of this transcriptional program in regulatory T cell biology.

Over the past three decades, Tregs have transitioned from a fundamental immunological concept to a therapeutic modality explored in autoimmunity, hematopoietic stem cell transplantation, and solid organ transplantation. Early preclinical studies demonstrated that adoptive transfer of regulatory T cells could prevent graft rejection, control autoimmunity, and mitigate graft-versus-host disease, paving the way for first-in-human clinical trials. These studies, primarily based on CD4^+^ Tregs, established feasibility and safety across multiple clinical settings and provided a foundation for further translational development.

Despite these advances, the clinical implementation of Treg-based therapies remains constrained by significant biological and technical challenges. Regulatory T cells are rare, phenotypically heterogeneous, and highly sensitive to inflammatory cues, necessitating carefully optimized strategies for isolation, expansion, stabilization, and quality control. In parallel, the field has diversified to include multiple regulatory populations and engineering approaches, including CD8^+^ regulatory T cells (1), antigen receptor–engineered Tregs, and stem cell–derived platforms, each introducing distinct manufacturing, mechanistic, and translational considerations. CD8^+^ Tregs are typically characterized by a CD8^+^CD45RC^low/-^ phenotype (2) and display transcriptional and functional programs distinct from conventional cytotoxic CD8^+^ T cells (3–6). Preclinical work has shown their efficacy in models of transplantation, autoimmunity, and neuroinflammation, culminating in the first clinical trial of CD8^+^ Tregs in kidney transplantation (7–9).

A key mechanistic distinction between CD4^+^ and CD8^+^ Tregs lies in their mode of antigen recognition. In transplantation, CD8^+^ Tregs benefit from long-lived donor MHC class I^+^ graft cells, which provide sustained and strong stimulation through direct, indirect, and semi-direct presentation pathways. In contrast, CD4^+^ Tregs rely primarily on donor MHC class II^+^ antigen-presenting cells, which are short-lived and thus only transiently sustain their activation. In autoimmune diseases, constitutive MHC-I expression by cells in inflamed organs may also sustain persistence of CD8^+^ Tregs along with inflamed antigen-presenting cells (APC). This difference may explain the enhanced durability of CD8^+^ Treg-mediated tolerance. Notably, to overcome this limitation, CD4^+^ Tregs are now being engineered with chimeric antigen receptors (CARs) recognizing donor MHC-I molecules, thereby enabling direct antigen-specific interactions with target tissues and potentially improving therapeutic efficiency.

In this context, a comprehensive and comparative analysis of Treg-based therapeutic strategies is required. This review focuses on the practical and mechanistic foundations of regulatory T cell therapy, including cell source selection, manufacturing workflows, stabilization of regulatory identity, and strategies to enhance specificity and persistence. Particular attention is given to the comparative features of CD4^+^ and CD8^+^ Tregs, as well as to emerging engineering and stem cell-based approaches, with the aim of clarifying key parameters that will shape the next generation of regulatory T cell therapies.

## 2 Autologous Treg therapy

Building on these biological insights and preclinical proofs of concept, the first translational efforts have focused on autologous Treg therapy, where cells are isolated from the patient, expanded *ex vivo* under good manufacturing practice (GMP) conditions, and reinfused to restore immune tolerance. This approach has the advantage of avoiding alloreactivity and graft rejection, and it has been the cornerstone of the earliest clinical trials in transplantation, GvHD, and autoimmunity. However, its success depends critically on three parameters: the purity of the isolated population, the ability to expand sufficient numbers while preserving function and stability, and the definition of an effective therapeutic dose.

### 2.1 Isolation of peripheral blood Tregs

The first critical step in autologous Treg therapy is the isolation of a sufficiently pure and viable regulatory population from peripheral blood of the patient. This is particularly challenging since Tregs represent only a small fraction of circulating lymphocytes and differ phenotypically between CD4^+^ and CD8^+^ subsets. Refining protocols for their enrichment is essential to meet clinical-grade manufacturing standards. Multiple approaches have therefore been developed, ranging from magnetic bead–based enrichment to high-resolution flow cytometry sorting, each with specific advantages and limitations.

Autologous Treg isolation begins with peripheral blood mononuclear cells (PBMCs) density gradient separation, followed by GMP-grade pre-enrichment via magnetic CD25^+^ selection (CliniMACS, Miltenyi Biotec) (10–12), which efficiently enriches both CD4^+^ and CD8^+^ Tregs (13). Specific selection strategies for CD4^+^ and CD8^+^ Treg subsets vary and are summarized in **Table 1**. However, magnetic bead-based selection restricts Treg isolation to highly expressed or absent markers and lacks the resolution to capture intermediate levels such as CD127^low^ or CD45RC^low/-^ expression, which are critical for precise Treg subsets definition and may compromise purity and stability. In contrast, flow cytometry-based sorting enables multiparametric gating with superior specificity for clinical-grade products, despite higher cost and the need for specialized expertise.

**Table 1:** Key parameters for isolation and expansion of CD4^+^ and CD8^+^ Tregs. .

Process step	Parameter	CD4^+^ Tregs	CD8^+^ Tregs	References
Isolation	Primary markers	CD25 ^hi^, CD127 ^low/-^	CD25^hi^, CD45RC ^low/-^	(10–13)
	Additional markers	CD45RA^+^, CCR7^+^, CD137^+^, CD154^-^	TNFR2^+^, GITR^+^, CD29^+^	(3, 14) NCT04817774
	Depletion	CD8^+^/CD19^+^	CD4^+^/CD19^+^	(15, 16)
Expansion	GMP media	X-VIVO 15 (Lonza), TexMACS (Miltenyi Biotec), CTS OpTmizer (ThermoFisher Scientific), Stem X-vivo (R&D) and SCGM (CellGenix)	X-VIVO 15 (Lonza), LymphoONE (Takara), ImmunoCult-XF (Stem Cell, Prime XV (Irvine Scientific), KBM541 (Kohjin Bio), Stem X-vivo (R&D) and SCGM (CellGenix)	(11, 16–20) NCT06777719
	Serum	2-5% human serum	Up to 10% human serum	(8, 16, 17, 21, 22) NCT06777719
	Key cytokines	IL-2 (500–3000 U/mL) + TGF-β (1–5 ng/mL)	IL-2 (25–1000 U/mL) + IL-15 (10 ng/mL)	(8, 16, 17, 22–26)
	Additives	Rapamycin (50–100 nM), ATRA	Rapamycin (50–100 nM)	(8, 16, 17, 21, 27–33)
	TCR stimulation	- Anti-CD3 antibodies (clone OKT3) + Anti-CD28 antibodies (clone CD28.2) - Anti-CD3/CD28 antibody-coated beads (MACS GMP ExpAct Treg Kit) or T Cell TransAct (Miltenyi Biotec) - Soluble tetramerized antibodies (ImmunoCult™ Human CD3/CD28 T Cell Activator, StemCell Technologies)		(8, 16, 17, 21)

Studies have demonstrated the feasibility of Tregs manufacturing from FACS-sorted CD25^+^CD127^−^ Tregs (34, 35). Today, the evolving GMP regulations make compliance with GMP standards a primary criterion for the production of these cells for clinical applications. This includes features such as effective aerosol containment, sterile sample handling capabilities, and validated software to ensure data integrity, traceability, and regulatory compliance. Several FACS platforms have been adapted or developed to meet these stringent requirements. Among them, the MACSQuant Tyto^®^ by Miltenyi Biotec stands out as a closed, microfluidic sorting system that avoids aerosol formation, significantly reducing contamination risks. It allows gentle, pressure-based sorting without droplet formation and is already widely used in GMP-compliant facilities for Treg, CAR-T, and natural killer (NK) cell isolation. The Sony SH800S, with its disposable microfluidic sorting chips, offers a flexible GMP-compatible option, suitable for smaller-scale or early-phase clinical trials. For more complex multiparametric sorting, the BD FACSymphony™ S6 and FACSAria™ Fusion can be adapted to GMP environments when installed in biosafety cabinets and paired with validated cleaning protocols. These instruments allow high-parameter cell analysis and sorting but require careful validation due to the open jet-in-air design. Thus, multiple technological pathways exist, each with trade-offs between flexibility, throughput, and biosafety.

Another critical factor that must be considered for the FACS-isolation of Tregs for autologous cell therapy is the ability to process the starting material efficiently, especially when dealing with large sample volumes or rare subsets. It is essential to estimate the total time required to isolate a sufficient number of target cells to initiate the manufacturing process, as prolonged sorting times can compromise cell viability and delay downstream steps. For rare populations like Tregs, which typically represent less than 1% of PBMCs, large blood volumes or leukapheresis are often necessary, and a pre-enrichment step such as magnetic bead-based selection may be necessary prior to FACS sorting. This not only improves sorting efficiency and throughput but also enhances the final purity of the product, ensuring that the isolation can be completed within a single working day.

Beyond the choice of markers and sorting platforms, several additional factors influence the feasibility of autologous Treg isolation for clinical use. While peripheral blood remains the most widely used source, alternative tissues such as umbilical cord blood (**Tables 2**, **3**) or pediatric thymic tissue (**Tables 4**, **5**) are also being explored (78, 86), though their availability is limited.

**Table 2:** Trial registry in AID.

NCT number	Study title	Disease	Source	Dose	Treg subtype	Phase	Enrolment	Start-end date	Status	Outcome
NCT03241784	T-Regulatory Cells in Amyotrophic Lateral Sclerosis	ALS	Autologous blood	1 x 10^6^/kg	CD4^+^CD25^+^	I	4	2016- 2018	Unknown	–
NCT04055623	Tregs in ALS	ALS	Autologous blood	1–3 x 10^6^/kg x6 infusions	CD4^+^CD25^+^FOXP3^+^ polyclonal	II	12	2019 - 2022	Unknown	Safe and well tolerated; 6/8 patients showed slow/no ALS progression (36)
NCT06169176	RAPA-501: Therapy of ALS Expanded Access Protocol	ALS	Autologous blood	2–8 x 10^7^ total, x4 infusions	FOXP3^+^, Th2 profile, stem-like T markers	I	?	2023 - *2026*	Available	–
NCT05695521	REGALS: Regulatory T Cells for ALS	ALS	Allogeneic (UCB-derived)	1 x 10^8^ total x9 infusions	CXCR3^+^ CXCR7^+^LFA1^+^ CD4^+^CD25^+^ (CK0803)	I	66	2023 - *2027*	Active, Not recruiting	–
NCT06671236	Tregs in the Treatment of Neurodegenerative Diseases	ALS	Autologous blood	1 x 10^6–^1 x 10^8^ x3 infusions	Polyclonal (NP001)	I	12	2024 - *2027*	Recruiting	–
NCT04220190	RAPA-501 Therapy for ALS	ALS	Autologous blood	8 x 10^7^ total x4 infusions	CD4^+^ and CD8^+^, GATA3^+^Th2 and FOXP3^+^ polyclonal	II/III	41	2025 - *2027*	Recruiting	–
EudraCT: 2014-004320-22	Administration of CD4^+^CD25^high^CD127^−^FOXP3^+^ Tregs for Relapsing-Remitting Multiple Sclerosis	MS	Autologous blood	4 x 10^7^/kg	CD4^+^CD25^high^CD127^−^FOXP3^+^ polyclonal	I/II	14	2014 - 2021	Unknown	Safe (37)
NCT06566261	ABA-101 in Participants with Progressive MS	MS	Autologous blood	Undisclosed	HLA-DRB1*15:01-specific TCR engineered FOXP3^+^	I	12	2024 - *2027*	Active, Not recruiting	–
NCT05214014	Treatment of Systemic Sclerosis With Autologous Tregs	Systemic sclerosis	Autologous blood	Undisclosed	CD4^+^CD25^+^	I/II	30	2022 - 2023	Unknown	+33% improvement vs standard therapy in skin elasticity, with no progression of skin thickening and enhanced ulcer healing at M6 (38)
NCT01210664	T1DM Immunotherapy Using CD4^+^CD127^lo/-^CD25^+^ Polyclonal Tregs	T1DM	Autologous blood	5 x 10^6^ - 2.6 x 10^9^ total	CD4^+^CD127^lo/-^CD25^+^ Polyclonal	I	16	2010 - 2017	Completed	Safe (18, 39, 40)
ISRCTN06128462	TregVac: Cellular therapy of type 1 diabetes with Tregs	T1D	Autologous blood	1–3 x 10^7^/kg	CD4^+^CD127^lo/-^CD25^+^ polyclonal	0	12	2011 - 2014	Completed	Safe, can prolong survival of β-cells (41, 42)
EudraCT: 2014-004319-35	TregVac 2.0: Therapy of type 1 diabetes with Tregs and anti-CD20 mAbs	T1D	Autologous blood	3 x 10^7^/kg x2 infusions	CD4^+^CD25^high^CD127^-^ polyclonal	I/II	36	2014 - 2022	Completed	No significant efficacy as monotherapy (43–45)
NCT02691247	Sanford/Lisata Therapeutics T-Rex Study Safety and Efficacy of CLBS03 in Adolescents With Recent Onset Type 1 Diabetes	T1D	Autologous blood	1 x 10^6–^2 x 10^7^/kg	CD4^+^CD25^hi^FOXP3^+^ polyclonal	II	113	2016 - 2020	Completed	Safe, no prevention of residual β cell function decline over 1 year (46)
NCT02772679	TILT T1DM Immunotherapy Using Polyclonal Tregs + IL-2.	T1DM	Autologous blood	3 x 10^6–^2 x 10^7^/kg	CD4^+^CD127^lo/-^CD25^+^ Polyclonal	I	16	2016 - 2021	Completed	Safe (39)
NCT06708780	Immunotherapy with Autologous Tregs in T1DM	T1DM	Autologous blood	Undisclosed	Undisclosed	I	20	2023 - *2027*	Recruiting	–
NCT06688331	PreTreg Treatment of Pre-diabetic State in Pediatric Population With Treg Cell Preparations and Rituximab	T1DM	Autologous blood	Undisclosed x2 infusions	CD4^+^CD25^+^CD127^-^	II	150	2025 - *2032*	Recruiting	–
NCT02932826	Safety Study and Therapeutic Effects of Umbilical Cord Blood Treg on Autoimmune Diabetes	T1DM	Allogeneic (UCB-derived)	1–5 x 10^6^/kg	polyclonal	I/II	40	2016 - *2025*	Recruiting	–
NCT03011021	Safety and Efficacy of Umbilical Cord Blood Regulatory T Cells Plus Liraglutide on Autoimmune Diabetes	T1DM	Allogeneic (UCB-derived)	1–5 x 10^6^/kg	CD4^+^ polyclonal	I/II	40	2017 - *2025*	Unknown	–
NCT02704338	Safety and Efficacy Study of Regulatory T Cells in Treating Autoimmune Hepatitis	AIH	Autologous blood	1–2 x 10^7^/kg	CD4^+^CD127^-^CD25^+^ Polyclonal	I/II	30	2016 - 2018	Unknown	Safe (47)
IRAS ID: 177127	AUTUMN Autologous T-regulatory cell tracking after InfUsion in AutoiMmuNe liver disease patients	AIH	Autologous blood	8.9 x 10^6^ - 8.9 x 10^7^ total	CD4^+^CD25^+^CD8^-^CD19^-^FOXP3^+^	0	4	2016 - 2017	Completed	Safe (48)
Eudract 2006-004712-44	CATS1Safety and Efficacy of Antigen-Specific Regulatory T-Cell Therapy for Patients With Refractory Crohn’s Disease	CD	Autologous blood	1 x 10^6–^1 x 10^9^ total	Antigen-specific (OVA) Tr1 IL10^+^ FOXP3^+^ CD25^+^ CD127^-^ CD62L^-^	I/II	20	2008 - 2011	Completed	Well tolerated at 10^6^, dose-related efficacy (49)
NCT02327221	Study Evaluating Ovasave, an Autologous Cell Therapy, in Patients With Active Crohn’s Disease	CD	Autologous blood	1 x 10^4–^1 x 10^7^ total x4 infusions	Ova-specific	II	32	2014 -2016	Terminated	Not conclusive
NCT03185000	TR004: TRIBUTE Treg Immunotherapy in Crohn’s Disease.	CD	Autologous blood	3–5 x 10^6^/kg	CD4^+^CD25^hi^CD127^lo^ CD45RA^+^	I	4	2022 - *2025*	Unknown	(50)
NCT04691232	ER-TREG01 Autologous Ex Vivo Expanded Regulatory T Cells in Ulcerative Colitis.	UC	Autologous blood	5 x 10^5–^1 x 10^7^/kg	CD4^+^CD25^+^CD127^−/lo^ polyclonal	I	11	2021 - 2023	Completed	Safe in 1st patient (51, 52)
NCT02428309	Autologous Polyclonal Tregs for Lupus	Lupus	Autologous blood	1 x 10^8^-1.6 x 10^9^ total	CD4^+^CD127^lo/-^CD25^+^ polyclonal	I	1	2015 - 2018	Terminated	Inconclusive due to recruitment limitations (1 patient) (53)
NCT03239470	DAIT NIAID Polyclonal Regulatory T Cells (PolyTregs) for Pemphigus	Pemphigus	Autologous blood	1-2.5 x 10^8^ total	CD4^+^CD25^+^CD127^lo/-^ polyclonal	I	5	2017 - 2023	Terminated	Terminated due to COVID-19; no SAEs
NCT05241444	CD4^LVFOXP3^ in Participants With IPEX	IPEX	Autologous blood	1 x 10^6–^1 x 10^7^/kg	LVFOXP3 LVCD271 CD4^+^	I	30	2022 - *2037*	Recruiting	–
NCT06201416	Single Doses of SBT777101 in Subjects With Rheumatoid Arthritis	RA	Autologous blood	undisclosed	Citrullinated-protein specific (CAR)-FOXP3^+^	I	24	2024 - *2026*	Recruiting	–

**Table 3:** Trial registry in HSC transplant.

NCT number	Study title	Disease	Source	Dose	Treg subtype	Phase	Enrolment	Start-end date	Status	Outcome
NCT00376519	Umbilical Cord Blood Treg Infusion Followed by Donor Umbilical Cord Blood Transplant in Treating Patients With High-Risk Leukemia or Other Hematologic Diseases	aGvHD	3^rd^-part UCB,4-6/6 matched	1 x 10^5–^3 x 10^6^/kg	CD4^+^CD25^+^ polyclonal on day -1 HSCT	I	3	2007 - 2008	Terminated/Slow accrual	Lower grade II–IV aGVHD; safety preserved (11, 54)
NCT00602693	T-Regulatory Cell Infusion Post Umbilical Cord Blood Transplant in Patients With Advanced Hematologic Cancer	aGvHD	3^rd^-part UCB, 4-6/6 matched -	1 x 10^5^ to 3 x 10^8^/kg	CD4^+^CD25^+^ polyclonal on day +1 HSCT	I	41	2007 - 2015	Completed	Lower grade II–IV aGVHD; safety preserved (11, 54)
NCT02423915	Fucosylated T Cells for GvHD Prevention	aGvHD	3^rd^-part UCB, 4-6/6 matched -	1–10 x 10^6^/kg	Fucosylated polyclonal on day -1 HSCT	I	5	2015 - 2020	Completed	1×10^6^/kg: tolerated; no infusion/engraftment effects; no aGVHD benefit; no cGVHD 1×10^7^/kg: not reported (55)
NCT02991898	Adoptive TReg Cell for Suppression of aGVHD After UCB HSCT for Heme Malignancies	aGvHD	3^rd^-part UCB, 4-6/6 matched	Treg: CD3 ratio 1:1 (<1.5 x 10^7^/kg)	CD4^+^CD25^+^ polyclonal Tregs on day 0 HSCT	II	3	2017 - 2019	Terminated/alternative technology for product	2/3 infusion toxicity; 72% chimerism (d+100); 2/3 grade II–IV aGVHD; no cGVHD (1y); 1/3 relapse; 2/3 OS (1y), 1/3 OS (2y); 3/3 infections
IS/11/6172/8309/8391	ALT-TEN Haploidentical-HSC transplanted patients treated with IL-10-anergized donor T Cells	GvHD	Matched allogeneic blood	1 x 10^5–^3 x 10^6^/kg	IL10^+^ CD4^+^ Tr1 on d+28 to +64	I/II	19	2000 - 2009	Completed	Safe; long-term disease-free survival in 4 patients (10^5^ IL-10 T cells) (56)
NKEBN/458-310/2008	First-in-man clinical trial on patients with graft versus host disease treated with human ex vivo expanded CD4^+^CD25^+^CD127^−^ Tregs	GvHD	Matched allogeneic blood	1 x 10^5–^3 x 10^6^/kg	CD4^+^CD25^+^CD127^−^ polyclonal	I	2	2005 - 2008	Terminated	Alleviated symptoms, reduced IS in cGvHD, transient aGVHD benefit (34)
EK 206082008	Adoptive transfer of allogeneic Tregs into patients with chronic graft-versus-host disease	cGvHD	Matched allogeneic blood	9.7 x 10^5^ - 4.45 x 10^6^/kg	CD4^+^CD25^+^CD127^lo^ FOXP3^+^ polyclonal on month >28 HSCT	I	5	2008 - 2012	Completed	Ameliored cGvHD, tapered IS, two cases of tumors (12, 21)
NCT01050764	BMT Protocol 204 Haploidentical Allogeneic Transplant With Post-transplant Infusion of Regulatory T-cells	GvHD prophylaxis	Matched allogeneic blood	1 x 10^5–^3 x 10^6^/kg	CD15^+^CD4^+^CD127^dim^ FOXP3^+^ on day 14–16 HSCT	I/II	10	2009 - 2014	Terminated due to covid-19.	Not conclusive.
EudraCT: 2012-000301-71	Donor Treg infusion (DTI) in patients with steroid-refractory chronic GVHD.	cGvHD	Allogeneic 9-10/10 matched blood	5 x 10^5^/kg	>1 month HSCT	II	35	2012 - 2022	Prematurely Ended	Low inclusion rate
NCT01660607	ORCA-T Phase 1–2 MAHCT w/T Cell Depleted Graft w/Simultaneous Infusion Conventional and Treg.	aGVHD	Allogeneic >9/10 matched blood	1–3 x 10^6^/kg	CD4^+^CD25^+^CD127^-^FOXP3^+^ polyclonal	I/II	68	2012 - 2023	Completed	Reduced GVHD; improved 1-yr GVHD-free relapse-free survival (57, 58)
NCT01911039	Phase 1 Infused Donor Tregs in Steroid Dependent/Refractory Chronic GVHD	cGvHD	Allogeneic >7/8 matched blood	1 x 10^5^ –1.5 x 10^6^/kg	Polyclonal Tregs	I	14	2013 - 2018	Completed	–
NCT01634217	Inducible Tregs (iTregs) in Non-Myeloablative Sibling Donor Peripheral Blood Stem Cell Transplantation	GvHD prophylaxis	Matched allogeneic blood	3 x 10^6–^3 x 10^8^/kg	CD4^+^CD25^+^FOXP3^+^ iTregs on day 0 HSCT	I	19	2013 - 2018	Completed	Safe (59)
NCT01903473	Donor Tregs Infusion in Patients With Chronic Graft-versus-host Disease (GVHD)	cGVHD	Allogeneic >9/10 matched blood	5 x 10^5^/kg	CD4^+^CD25^+^FOXP3^+^ polyclonal on month >1 HSCT	II	19	2013 - 2022	Terminated	Slow recruitment
EudraCT: 2012-002685-12	Treatment of steroid resistant severe acute gastrointestinal GVHD with *in vitro* expanded donor-derived Tregs	aGvHD	Allogeneic blood	5 x 10^6^/kg	CD4^+^CD25^+^FOXP3^+^ polyclonal (Treg002)	II	29	2013 - 2024	Prematurely Ended	Increased Treg pool of several patients (60)
NCT01937468	Trial of Tregs Plus Low-Dose Interleukin-2 for Steroid-Refractory Chronic GVHD	cGvHD	Allogeneic blood	1 x 10^5–^1 x 10^6^/kg	Polyclonal on week >4 HSCT	I	25	2013 - 2025	Active, Not recruiting	Safe (61)
NCT01795573	Ex-vivo Expanded Donor Tregs for Prevention of Acute GVHD	aGvHD	Matched allogeneic blood	Undisclosed	Recipient-Ag specific on day -2 HSCT	I	38	2014 - 2020	Completed	–
NCT03977103	Irradiation-based Myeloablative Conditioning Followed by Treg/Tcon Immunotherapy in HSCT	cGVvHD	Matched allogeneic blood	2 x 10^6^/kg	Non expanded polyclonal on day -4 HSCT	II	80	2014 - 2023	Unknown	Moderate/severe cGVHD/relapse-free survival was 75% after 29 months (10, 62, 63)
NCT02385019	TREGeneration A Phase 1/2 Trial of Donor Tregs for Steroid-Refractory Chronic GVHD	cGvHD	Allogeneic blood	5 x 10^5–^3 x 10^6^/kg	CD8^-^CD19^-^CD25^+^ polyclonal on day >28 HSCT	I/II	22	2015 - 2019	Unknown	–
NCT02749084	Multiple Donor Treg DLI for Severe Refractory Chronic GVHD	cGvHD	Mismatched allogeneic blood	5 x 10^5–^2 x 10^6^/kg	CD8^-^CD19^-^CD4^+^CD25^+^CD127^lo-^ polyclonal on month >1 HSCT	I/II	18	2016 - 2022	Completed	–
NCT06551584	ORCA-T Phase I Trial for Patients w/o Advanced Hematologic Malignancies Undergoing Allogeneic HCT	GvHD prophylaxis	Allogeneic 7/8 matched blood	3 x 10^6^/kg	CD4^+^CD25^+^CD127^lo/-^FOXP3^+^CD19^-^CD56^-^CD14^-^ on day 0 HSCT	I	24	2019 - *2027*	Not yet recruiting	–
NCT03802695	ORCA-Q A Phase 1 Study of Orca-Q in Recipients Undergoing Allogeneic Transplantation for Hematologic Malignancies	GvHD prophylaxis	Allogeneic 7-8/8 matched blood	Undisclosed	CD4^+^CD25^+^CD127^lo/-^FOXP3^+^CD19^-^CD56^-^CD14^-^	I	300	2019 - *2027*	Recruiting	–
NCT04013685	Precision-T: A Study of Orca-T in Recipients Undergoing Allogeneic Transplantation for Hematologic Malignancies	GvHD prophylaxis	Matched allogeneic blood	Undisclosed	CD4^+^CD25^+^CD127^lo/-^FOXP3^+^CD19^-^CD56^-^CD14^-^ on day 0 HSCT	III	255	2019 - *2027*	Active, Not recruiting	–
NCT05088356	Reduced Intensity Allogeneic HCT in Advanced Hematologic Malignancies w/T-Cell Depleted Graft	GvHD	Allogeneic 7-8/8 matched blood	1–3 x 10^6^/kg	Polyclonal on day 0 HSCT	I	60	2021 - *2025*	Recruiting	–
NCT04640987	Stem Cell Transplant From Donors After Alpha Beta Cell Depletion in Children and Adults With T-allo10 Cells Addback	GvHD prophylaxis	Mismatched allogeneic blood	1 x 10^5–^1 x 10^6^/kg	Recipient CD14-amplified CD4^+^T cells Tr1 on d+35	I	22	2021 - *2029*	Recruiting	Safe, improved early IR and reduced GvHD risks (64)
NCT05322850	Phase I/II Trial: Engineered Donor Graft (Orca Q) for Pediatric Hematopoietic Cell Transplant (HCT)	GvHD prophylaxis	Allogeneic 7-8/8 matched blood	Undisclosed	CD4^+^CD25^+^CD127^lo/-^FOXP3^+^CD19^-^CD56^-^CD14^-^	I/II	40	2022 - *2026*	Recruiting	–
NCT05316701	Precision-T: A Randomized Study of Orca-T in Recipients Undergoing Allogeneic Transplantation for Hematologic Malignancies	GvHD prophylaxis	Matched allogeneic blood	3 x 10^6^/kg	CD4^+^CD25^+^CD127^lo/-^FOXP3^+^CD19^-^CD56^-^CD14^-^ on day 0 HSCT	III	187	2022 - *2026*	Active, Not recruiting	–
NCT05507827	Myeloablative Conditioning Orca-T & Allogeneic Donor-Derived CD19/CD22-CAR TCells in B-Cell ALL	GvHD prophylaxis	Matched allogeneic blood	Undisclosed	CD4^+^CD25^+^CD127^lo/-^FOXP3^+^CD19^-^CD56^-^CD14^-^ polyclonal on day 0 HSCT	I	22	2022 - *2026*	Active, Not recruiting	–
NCT05095649	Treg4GVHD Donor Tregs for cGVHD in Patients Who do Not Obtain Complete Remission With Ruxolitinib	GVHD	Allogeneic blood	2 x 10^6^/kg	CD25^hi^CD8^-^CD19^-^ FOXP3^+^ on week>12 HSCT	II	15	2022 - *2026*	Recruiting	–
NCT06462365	Prevention of GvHD in Participants With Hematological Malignancies Undergoing HSCT	GvHD prophylaxis	Mismatched allogeneic blood	>2.5 x 10^7^ total	LV^IL10+^ Tr1 after HSCT (TRX103)	I	36	2024 - *2027*	Recruiting	Safe at 2.5x10^7^ (65)
NCT06195891	Orca-T Following Chemotherapy and Total Marrow and Lymphoid Irradiation for the Treatment of AML, ALL or Myelodysplastic Syndrome	GvHD prophylaxis	Matched allogeneic blood	Undisclosed	Partially Engineered Treg Donor Graft (TRGFT-201) on day 0 HSCT	I	33	2024 - *2027*	Recruiting	–
NCT05993611	Allogeneic CD6 Chimeric Antigen Receptor Tregs (CD6-CAR Tregs) for the Treatment of Patients With Chronic GvHD After Allogeneic Hematopoietic Cell Transplantation	cGvHD	Matched allogeneic blood	Undisclosed. Dose escalation	CD6^-^CAR CD4^+^ CD25^high^ CD127^low^ FOXP3^+^ on day >60 HSCT	I	27	2024 - *2028*	Recruiting	–
NCT06920199	Treatment of Refractory cGVHD by Donor-derived Treg Cell Injection Combined With Recombinant Human Interleukin-2	cGVHD	Allogeneic blood	1 x 10^6–^1 x 10^7^/kg	CD4^+^Tregs on week >4 HSCT	EARLY_I	18	2025 - *2027*	Recruiting	–
NCT06991361	EVENT Ex Vivo-Expanded Tregs Plus Low-Dose Interleukin-2 for Steroid-Refractory Chronic GVHD	cGvHD	Allogeneic >7/8 matched blood	3 doses, undisclosed	CD4^+^ CD25^high^ CD127^low^ FOXP3^+^ (EVE-Treg) on day >28 HSCT	I	21	2025 - *2028*	Not yet recruiting	–
NCT06411184	Safety and Efficacy of Treg Cell in the Treatment of GVHD	GVHD	Matched autologous blood	1–8 x 10^6^/kg	CD4^+^ on week >4 HSCT	I/II	20	2024 - *2027*	Not yet recruiting	–

**Table 4:** Trial registry in SOT.

NCT number	Study title	Disease	Source	Dose	Treg subtype	Phase	Enrolment	Start-end date	Status	Outcome
NCT01446484	RSMU-001 Treatment of Children With Kidney Transplants by Injection of CD4^+^CD25^+^FOXP3^+^ T Cells to TASKp Prevent Organ Rejection	Kidney Transplant	Autologous blood	2 x 10^8^ total x2 infusions	CD4^+^CD25^+^CD127^lo^ FOXP3^+^ on days +30 and +180 SOT	I/II	30	2011 - *2014*	Unknown	–
NCT02088931	Treg Adoptive Therapy for Subclinical Inflammation in Kidney Transplantation.	Kidney Transplant	Autologous blood	5 x 10^6–^1 x 10^7^/kg	CD4^+^CD127^lo/-^CD25^+^ Polyclonal month +6	I	3	2014 - 2016	Completed	Safe (19)
NCT02145325	Trial of Adoptive Immunotherapy With TRACT to Prevent Rejection in Living Donor Kidney Transplant Recipients	Kidney Transplant	Autologous blood	5 x 10^8–^5 x 10^9^ total	CD4^+^CD25^+^FOXP3^+^ on month +2 SOT	I	10	2014 - 2016	Completed	Safe (31)
NCT02091232	The ONE Study Infusion of T-Regulatory Cells in Kidney Transplant Recipients	Kidney Transplant	Autologous blood	3–8 x 10^9^ total	Donor Ag specific CD4^+^CD25^hi^CD127^lo^ on day +7 SOT	I	4	2014 - 2016	Completed	Safe (66, 67)
NCT02129881	The ONE Study UK Treg Trial.	Kidney Transplant	Autologous blood	1–10 x 10^6^/kg	CD4^+^CD127^lo/-^CD25^+^ Polyclonal on day +5 SOT	I/II	15	2014 - 2017	Completed	Safe, reduced IS (16, 66, 68)
NCT02371434	The ONE Study nTreg Trial	Kidney Transplant	Autologous blood	5 x 10^5–^3 x 10^6^/kg	CD4^+^CD25^+^FOXP3^+^ polyclonal on day +7 SOT	I/II	17	2015 - 2017	Completed	Safe, tapered IS (66, 69)
NCT02244801	Donor-Alloantigen-Reactive Treg (darTreg) Therapy in Renal Transplantation	Kidney Transplant	Autologous blood	3–8 x 10^9^ total	Donor Ag specific CD4^+^CD25^hi^CD127^lo^ after SOT	I	6	2015 - 2018	Completed	Safe (20, 66)
NCT02711826	TASK Treg Therapy in Subclinical Inflammation in Kidney Transplantation.	Kidney Transplant	Autologous blood	1 x 10^8–^1 x 10^9^ total	CD4^+^CD25^+^CD127^lo^ polyclonal	I/II	32	2016 - 2023	Completed	–
ISRCTN11038572	TWO study: cell therapy trial in renal transplantation	Kidney Transplant	Autologous blood	5 x 10^6–^1 x 10^7^/kg	CD4^+^CD25^+^FOXP3^+^ polyclonal (TR001) on day +5 SOT	II	68	2016 - *2031*	Recruiting	Preliminary results from 7 patients showed 100% survival and no acute rejection at 18 months (16, 70)
NCT03284242	A Pilot Study Using Autologous Regulatory T Cell Infusion Zortress (Everolimus) in Renal Transplant Recipients	Kidney Transplant	Autologous blood	1 x 10^8–^1 x 10^9^ total	CD25^+^ polyclonal	EARLY_I	9	2019 - 2024	Completed	Safe (71, 72)
NCT04817774	STEADFAST Safety & Tolerability Study of Chimeric Antigen Receptor T-Reg Cell Therapy in Living Donor Renal Transplant Recipients.	Kidney Transplant	Autologous blood	1 x 10^4–^1 x 10^9^/kg	Donor HLA-A2-CAR CD4^+^CD45RA^+^CD25^+^ CD127^lo/-^FOXP3^+^ TX200-TR101, week >2 SOT	I/II	26	2021 - *2025*	Active, Not recruiting	–
NCT06777719	Eight-Treg Study: Trial of Adoptive Immunotherapy With Autologous ex Vivo Expanded Regulatory CD8+ T Cells in Living Donor Kidney Transplant Recipients	Kidney Transplant	Autologous blood	1–8 x 10^6^/kg	CD8^+^CD4^-^CD56^-^CD45RC^low/-^ polyclonal on day -1 SOT	I	9	2025 - *2027*	Not yet recruiting	–
NCT06552169	TRACT-KD-101/RETIRE (TRK-001) Treg Therapy to Achieve Immunosuppression REduction	Kidney Transplant	Autologous blood	Undisclosed	CD4^+^ CD25^high^ CD127^low^ FOXP3^+^ on day +53 to +67 SOT	II	34	2025 - *2031*	Recruiting	–
NCT03943238	TLI, TBI, ATG & HSC Transplantation and Recipient T Regs Therapy in Living Donor Kidney Transplantation	Kidney + HSC Transplant (same donor)	Autologous blood	>2.5 x 10^7^/kg	polyclonal on day +1 HSCT, + 14 of SOT	I	22	2019 - 2023	Recruiting	1/2 persistent multilineage mixed chimerism; 1/2 pre/post-transplant Tcon/Treg expansion; 1/2 persistent alloreactive clones that may contribute homeostatic mixed chimerism and donor tolerance (73)
NCT03867617	Cell Therapy for Immunomodulation in Kidney Transplantation	Kidney + HSC Transplant (same donor)	Autologous blood	3 x 10^6^ - 1.5 x 10^7^/kg	CD45RA^+^CD4^+^CD25^hi^CD127^lo/-^ polyclonal (Trex001) on day +3 SOT	I/II	12	2019 - *2028*	Active, Not recruiting	(74)
UMIN-000015789	A Pilot Study of Operational Tolerance With a Treg-Based Cell Therapy in Living Donor Liver Transplantation	Liver Transplant	Autologous blood	8.3 x 10^6^ - 4.6 x 10^7^/kg	Enriched in donor Ag CD4^+^CD25^+^FOXP3^+^	I/II	10	2010 - 2012	Completed	Safe, effective for drug minimization and operational tolerance induction (75)
NCT01624077	Safety Study of Using Tregs Induce Liver Transplantation Tolerance	Liver Transplant	Autologous blood	9 x 10^7–^5 x 10^8^ total	Donor Ag specific CD4^+^CD25^+^CD127^-^ from M + 2 to Y + 1 SOT	I	1	2014 - 2015	Unknown	–
NCT02166177	ThRIL Safety and Efficacy Study of Treg Therapy in Liver Transplant Patients	Liver Transplant	Autologous blood	5 x 10^5^ – 4.5 x 10^6^/kg	CD4^+^CD25^hi^ polyclonal (TR002) on month +3 to 12 SOT	I/II	9	2014 - 2018	Completed	Safe; transient Treg increase; reduced anti-donor T-cell responses (17, 76)
NCT02188719	deLTa Donor-Alloantigen-Reactive Treg (darTregs) in Liver Transplantation	Liver Transplant	Autologous blood	2.5 x 10^7^ - 9.6 x 10^8^ total	Donor-Ag specific CD4^+^CD25^hi^CD127^lo^	I	15	2014 - 2019	Terminated	Safe, could not be completed within the grant timeline
NCT02474199	ARTEMIS Donor Alloantigen Reactive Tregs (darTregs) for Calcineurin Inhibitor (CNI) Reduction.	Liver Transplant	Autologous blood	3–5 x 10^8^ total	Donor-Ag specific CD4^+^CD25^+^CD127^lo/−^ on year +3 SOT	I/II	15	2016 - 2019	Completed	Safe; no AEs; 2/5 CNI ↓75% with stable LFTs (>12 w); generalized Treg activation/senescence; reduced donor reactivity (77)
NCT03577431	LITTMUS-MGH Liver Transplantation With Tregs at MGH	Liver Transplant	Autologous blood	1 x 10^6^ - 1.25 x 10^8^ total	Donor Ag CD4^+^CD25^+^CD127^lo^ (arTreg-CSB) on week >52 SOT	I/II	9	2019 - *2027*	Active, Not recruiting	–
NCT03654040	LITTMUS-UCSF Liver Transplantation With Tregs at UCSF	Liver Transplant	Autologous blood	9 x 10^7–^5 x 10^8^ total	Donor Ag CD4^+^CD25^+^CD127^lo^	I/II	42	2021 - 2023	Terminated	Terminated before dosing (no participants treated)
NCT05234190	LIBERATE Safety and Clinical Activity of QEL-001 in A2-mismatch Liver Transplant Patients	Liver Transplant	Autologous blood	Undisclosed	HLA-A2-CAR-CD4^+^CD25^high^CD127^low^FOXP3^+^ on y+1 to +5 SOT	I/II	33	2022 - *2040*	Recruiting	–
NCT04924491	THYTECH Cell Therapy With Treg Cells Obtained From Thymic Tissue (thyTreg) to Prevent Rejection in Heart Transplant Children	Heart Transplant	Autologous thymus	1–2 x 10^7^/kg	CD25^+^FOXP3^+^CD4^+^ CD8^het^	I/II	11	2020 - *2026*	Recruiting	Safe (78, 79)
NCT03162237	Safety and Efficacy Study of Islets Xenotransplantation	Islet xeno-transplant	Autologous blood	2 x 10^6^/kg	Polyclonal	NA	20	2013 - 2018	Completed	No PERV transmission (10/10); improved clinical status; higher transplant score vs Japanese study (80, 81)
NCT04820270	AutoTregIsl Infusion of Autologous T Reg at the Time of Transplantation of Allogenic Islets of Langerhans	Islet Transplant	Autologous blood	Undisclosed	Polyclonal, on day 0 Tx	I	8	2018 - 2021	Unknown status	Safe (82)
NCT03444064	PolyTreg Immunotherapy in Islet Transplantation	Islet Transplant	Autologous blood	4–6 x 10^8^ total	CD4^+^CD25^+^CD127^lo/-^ polyclonal on week +6 post Tx	I	11	2018 - 2024	Completed	–

**Table 5:** Trial registry in inflammatory diseases.

NCT number	Study title	Disease	Source	Dose	Treg subtype	Phase	Enrolment	Start-end date	Status	Outcome
NCT04482699	RAPA-501-Allo Therapy of COVID-19-ARDS	Severe COVID-19 Disease	Allogeneic blood	4 x 10^7^ - 1.6 x 10^8^ total	CD4^+^ and CD8^+^, GATA3^+^Th2 and FOXP3^+^ polyclonal	I/II	1	2020 - 2021	Terminated	Change in eligible patient population
NCT04468971	RESOLVE Regulatory T Cell infuSion fOr Lung Injury Due to COVID-19 PnEumonia	COVID-19 ARDS	Allogeneic (UCB-derived)	1–3 x 10^8^ total	CD4^+^CD25^+^CD127^lo^ FOXP3^+^ polyclonal (CK0802)	I	45	2020 - 2021	Completed	Safe (83)
NCT06052436	THYTECH2 Cell Therapy With Treg Cells Obtained From Thymic Tissue (thyTreg) to Control the Immune Hyperactivation Associated With COVID-19 and/or Acute Respiratory Distress Syndrome	Systemic Inflammatory Response Syndrome	Allogeneic thymus	5 x 10^6–^1 x 10^7^/kg	CD25^+^FOXP3^+^CD4^+^ CD8^het^	I/II	24	2023 - *2027*	Recruiting	–
NCT03773393	A Clinical Trial of CK0801 (a New Drug) in Patients With Bone Marrow Failure Syndrome (BMF)	Bone Marrow Disease	Allogeneic (UCB-derived)	1 x 10^6–^1 x 10^7^/kg	CD4^+^CD25^+^	I	18	2019 - *2027*	Active, Not recruiting	Safe, hints of efficacy in 3/4 patients (84)
NCT05423691	LIMBER-TREG108 Leading in MPNs Beyond Ruxolitinib in Combo With T-Regs.	Bone Marrow Disease - Myelofibrosis	Allogeneic (UCB-derived)	1 x 10^8^ total x6 infusions	CXCR4^+^ CD4^+^CD25^+^ CK0804	I	24	2022 - *2026*	Recruiting	Safe, improved symptoms (85)
NCT03865017	A Safety and Tolerability Study of GB301	Alzheimer Disease	Autologous blood	1.7 x 10^5^/kg	CD4^+^CD25^+^ polyclonal	I/II	20	2019 - 2021	Unknown	–
NCT06361836	Study of Single Doses of SBT777101 in Subjects With Hidradenitis Suppurativa	Hidradenitis Suppurativa	Autologous blood	Undisclosed	Citrullinated-protein specific (CAR)-FOXP3^+^	I	24	2024 - *2026*	Recruiting	–

For CD4^+^ Tregs, multi-step purification strategies are usually required to achieve high purity from the outset. In contrast, CD8^+^ Treg isolation typically starts from a broader CD8^+^CD45RC^low/-^ fraction, which is less pure initially but undergoes strong selective enrichment during the expansion phase, ultimately yielding a stable and highly suppressive population. A persistent challenge is the risk of contaminating effector T or Natural Killer (NK) cells within the final product, underscoring the importance of stringent multi-step purification and culture conditions. Ultimately, the reproducibility and scalability of isolation protocols, together with their compliance with GMP standards, are decisive for successful translation of both CD4^+^ and CD8^+^ Treg therapies into the clinic. As with CD4^+^ Tregs, GMP-compatible closed systems such as the Tyto^®^ or microfluidic sorters are necessary to allow the safe and scalable manufacture of CD8^+^ Tregs for clinical applications. These advances now enable the first-in-human trials of autologous CD8^+^ Treg therapy.

In summary, whether targeting CD4^+^ or CD8^+^ Tregs, isolation strategies must balance yield, purity, and practicality, often combining pre-enrichment, multiparametric sorting, and culture-driven stabilization. Yet, obtaining a pure population is only the first step, successful therapy also requires expanding these rare cells to clinically relevant numbers while maintaining their regulatory phenotype and suppressive function.

### 2.2 *Ex vivo* expansion

Because Tregs are rare in peripheral blood, expansion is essential to obtain therapeutic doses. This step not only increases cell numbers but also acts as a functional filter: culture conditions can stabilize FOXP3 expression, suppress contaminating effector cells, and reinforce the regulatory program. For CD4^+^ Tregs, expansion protocols are relatively mature and have already been translated into clinical trials. In contrast, CD8^+^ Tregs are typically isolated in a broader, less pure fraction, but their selective growth advantage under defined conditions allows robust enrichment during expansion, ultimately yielding a stable regulatory product. The translation of culture conditions to clinical-grade protocols is often a lengthy and labor-intensive process. Multiple parameters, including medium composition, serum supplementation, cytokine cocktails, metabolic or pharmacologic modulators, and T cell receptor (TCR) stimulation, must therefore be carefully optimized to ensure both scalability and functional stability (**Table 1**). Cytokine requirements diverge markedly by lineage and subset: CD4^+^ Tregs depend on interleukin-2 (IL-2), often combined with transforming growth factor beta (TGF-β) and in some protocols, interleukin-10 (IL-10), whereas CD8^+^CD45RC^low/-^ Tregs benefit from IL-2 and interleukin-15 (IL-15). Rapamycin enhances FOXP3 stability across both CD4^+^ and CD8^+^ Treg lineages by inhibiting the mammalian target of rapamycin (mTOR) pathway, a key regulator of effector T cell differentiation and metabolism (16, 17, 21, 27–31). This selective mTOR inhibition suppresses effector T cells while boosting CD8^+^ Treg suppressive capacity (2.5-fold) and proliferation (3-fold) (8). All-trans retinoic acid (ATRA) promotes a more stable FOXP3^+^ phenotype in CD4^+^ Tregs and increases the expression of gut-homing markers, thereby improving both functionality and tissue-targeting potential of expanded Tregs (30, 32, 33). Modulating metabolic pathways through PI3K/AKT targeting (87), and epigenetic modulators (such as DNMT inhibitors) (88, 89) are also under investigation. Serum supplementation also remains an important parameter for large-scale expansion. However, autologous plasma is limited by volume constraints, disease-related alterations, and batch variability, driving development of xeno-free GMP serum replacements (i.e. CTS Immune Cell SR) and purified human serum albumin as standardized Treg culture supplements.

In practice, *ex vivo* protocols can achieve 100- to 1,000-fold expansion within 2–3 weeks, starting from a low purity fraction that progressively enriches during culture to reach >90% purity and strong suppressive function. Quality control during expansion is essential, including monitoring of key markers expression such as FOXP3 expression and Treg-specific demethylated region (TSDR) demethylation, suppressive activity *in vitro*, and cytokine profiles, to ensure lineage stability.

Importantly, stable Treg identity is closely linked to epigenetic imprinting at the FOXP3 locus, particularly the TSDR, where extensive demethylation is associated with durable lineage commitment, whereas partially methylated populations may retain plasticity (90). A major concern remains the risk of phenotypic drift or lineage instability, particularly under inflammatory conditions. Pro-inflammatory cytokines such as IL-6 and IL-1β can promote downregulation or loss of FOXP3 expression and drive functional reprogramming toward effector phenotypes. This plasticity may result in acquisition of Th17-like features in CD4^+^ Tregs (91) or cytotoxic programs in CD8^+^ Tregs (92), particularly in environments rich in inflammatory signals. In addition, heterogeneity within expanded products, including variable TSDR methylation states, may contribute to differential stability and functional persistence after transfer.

Notably, thymus-derived natural Tregs (nTregs) exhibit greater epigenetic stability and sustained FOXP3 expression compared with peripherally induced Tregs (iTregs), which are more prone to instability and cytokine-driven reprogramming (90). This distinction has important implications for manufacturing strategies and clinical outcomes. Similarly, emerging sources such as iPSC-derived Tregs offer scalability advantages but raise concerns regarding the fidelity of epigenetic programming and long-term lineage stability, which remain under active investigation.

These risks are mitigated by optimized culture conditions, including rapamycin, ATRA, and cytokine cocktails. Notably, CD4^+^ Tregs favor high-dose IL-2, TGF-β, and strong CD3/CD28 stimulation, whereas CD8^+^ Tregs expand optimally with IL-2/IL-15, rapamycin, and lower-intensity signals (8).

Despite these advances, evidence from early clinical studies (**Tables 2**-**5**) suggests that maintaining phenotypic and functional stability *in vivo* remains a critical challenge, underscoring the need for improved biomarkers of lineage fidelity and long-term persistence. These advances now allow the reproducible production of billions of clinical-grade Tregs under GMP conditions, paving the way for their evaluation in early-phase clinical trials across transplantation and autoimmunity.

### 2.3 Effective dose

Having established protocols that enable the large-scale expansion of stable CD4^+^ and CD8^+^ Tregs under GMP conditions, the next critical question is how many cells are required to achieve therapeutic efficacy *in vivo*.

The precise dose needed to promote immune tolerance remains difficult to define, and is closely related to the time of administration, ideally before inflammatory processes or effector cell activation to limit tissue and organ damages.

Two main approaches can be used to estimate the effective dose of Tregs for therapy. The first relies on determining the proportion of Tregs required to promote immune tolerance, while the second uses allometric scaling from mouse models. Compared to small-molecule drugs, scaling up immune cell therapies is often more straightforward and predictable, as the pharmacokinetics and pharmacodynamics of immune cells, such as trafficking, renewal, and dose-response behaviors, are less affected by interspecies differences in body mass and metabolism (93–96).

Data from mouse studies suggest that preventing transplant rejection would require either 33% CD4^+^ Tregs or 60% CD8^+^ Tregs among total T cells (8, 96). Given that an adult human harbors approximately 3 × 10¹¹ T cells (97, 98), this translates to roughly 8.4 × 10¹^0^ CD4^+^ Tregs or 1.75 × 10¹¹ CD8^+^ Tregs, doses far beyond what is currently clinically feasible. Focusing solely on the circulating blood compartment, which contains around 5 × 10^9^ T cells (99), the required dose would still be about 1.4 × 10^9^ CD4^+^ Tregs or 2.9 × 10^9^ CD8^+^ Tregs. These high numbers raise significant safety and manufacturing challenges.

Alternatively, *in vitro* suppression assays provide dose estimates based on Treg-to-effector T cell ratios required for 50% suppression: approximately 1:16 for CD4^+^ Tregs (31) and 1:3 for CD8^+^ Tregs (8). Based on these ratios, effective doses might be around 2.25 × 10^6^ CD4^+^ Tregs/kg or 1.2 × 10^7^ CD8^+^ Tregs/kg to control inflammation. These values, although still approximations, more closely align with doses currently explored in clinical trials.

Initial clinical trials using CD4^+^ Tregs in humans started with doses ranging from 1 × 10^5^ CD4^+^ Tregs/kg and proved safety up to 3 × 10^8^ CD4^+^ Tregs/kg (54, 59) (NCT00602693, NCT01634217) or total doses up to 2.6 x 10^9^ (18, 39, 40, 49) (NCT01210664, Eudract 2006-004712-44) (**Table 6**). The ongoing Eight Treg Study (NCT06777719) plans to evaluate escalating doses of CD8^+^ Tregs from 1 to 8 × 10^6^ cells/kg. Though these clinical doses may appear low relative to theoretical estimates, Tregs are expected to expand *in vivo* and exert broader immunoregulatory effects, including the modulation of other immune cell populations. Furthermore, their use is often combined with immunosuppressive agents such as corticosteroids to enhance efficacy in clinical settings.

**Table 6:** Synthesis of trials.

Domain	Design/control	Cohort size	Primary endpoint robustness	Concomitant IS/confounding	Immunomonitoring/persistence	Durability of benefit
ALS	Phase I–II, open-label, single-arm, repeated infusions	Very small–small (≈4–60).	Safety, disease progression rate	Standard ALS care varies; no controlled pharmacologic IS	Peripheral Treg counts and function; limited long-term persistence data.	Preliminary signals of slowed disease progression
T1D/other AID	Phase I–II, mostly open-label safety trials; frequent adjunct therapies.	Small–moderate (≈10–150).	Safety, C-peptide preservation, β-cell function	Combinations with IL-2/anti-CD20 blur Treg-specific effects.	Detailed phenotyping and function; Tregs seen for weeks–months, variable durable persistence.	Transient C-peptide preservation; durable remission/insulin independence not consistently achieved.
IBD (Crohn’s/UC)	Phase I–II, mostly open-label; some early terminations.	Small (≤40, often split by dose).	Safety; clinical indices mostly exploratory.	Steroids/biologics frequently adjusted during trial, heavily confounding efficacy.	Limited persistence studies	some dose-related efficacy signals
HSCT/GvHD	Phase I-III, single-arm pilots & multi-center randomized donor-graft studies.	Very small (pilot) to large (≥150–300) in engineered graft programs.	From safety only to robust composite endpoints (GVHD-free/relapse-free survival)	Standard transplant immunosuppression, heterogeneous prophylaxis and donor/graft factors are major confounders.	Rich monitoring (chimerism, Treg/Tcon, TCR-seq, CAR/TCR tracking); evidence for months–years skewing toward Tregs in some programs.	Reduced GVHD and better GVHD-free/relapse-free survival beyond 1–2 years in some programs.
SOT (kidney/liver/islet/heart)	Phase I–II, mostly open-label, single-arm; a few structured consortia designs.	Small–moderate (≈4–70 per trial).	Safety; rejection, graft function and IS reduction as exploratory efficacy	Layered on standard CNI-based IS; planned IS minimization makes attribution to Tregs difficult.	Counts & donor-specific functional assays; persistence beyond months is heterogeneous	Some successful drug minimization, Early signals of operational tolerance
Systemic inflammatory/other	Phase I–II, single-arm or dose-escalation	Very small–small (≈1–45).	Safety and inflammatory markers	High-dose steroids and evolving standards (e.g., COVID-19) create major temporal and treatment confounding.	Basic Treg and cytokine monitoring; follow-up usually limited to weeks–few months.	Early signals of safety, Short-term improvements hard to distinguish from natural course and co-treatments; no established durable effect.

Altogether, defining the “effective dose” of Tregs is not only a matter of cell numbers but also of timing, persistence, and context. Administration prior to effector T cell activation appears most favourable, as inflammation can both limit Treg function and cause irreversible tissue damage. While theoretical calculations suggest that very high doses might be required, in practice CD8^+^ Tregs display greater per-cell suppressive potency than CD4^+^ Tregs (100–104), potentially reducing the number needed to achieve therapeutic benefit. Moreover, the ability of infused Tregs to proliferate *in vivo*, survive long-term, and traffic efficiently to target tissues may compensate for relatively modest infused doses. Importantly, dose escalation must also balance safety, since excessive Treg numbers could impair protective immunity against pathogens or tumors.

For these reasons, early-phase trials adopt conservative dosing and focus on biological readouts of efficacy, such as persistence, lineage stability, cytokine modulation, and tissue homing, rather than absolute infused cell counts. Ultimately, the effective dose is likely to be disease- and context-specific, guided by a combination of quantitative modelling and real-time immunomonitoring in patients.

While autologous Treg therapies have demonstrated feasibility, their scalability, cost, and patient-to-patient variability remain significant barriers. These challenges have spurred the development of allogeneic strategies, aiming to provide standardized, off-the-shelf Treg products that can overcome the limitations of individualized manufacturing.

## 3 Allogeneic Treg therapy

To address the inherent limitations of autologous approaches, including low yield, long manufacturing times, and variability in cell quality, researchers are now exploring allogeneic Treg therapies. In this strategy, regulatory T cells are derived from healthy donors, offering access to a virtually unlimited source of starting material and enabling the creation of standardized, “off-the-shelf” products. However, because allogeneic Tregs are naturally recognized and rejected by the recipient’s immune system, their clinical application requires strategies to overcome immunogenicity. Advances in gene editing, HLA/MHC silencing, and immune-evasive engineering are making it possible to generate allogeneic Treg products that can persist and function across HLA barriers. This section reviews the current state of donor-derived and engineered allogeneic Treg therapies, highlighting their potential and the challenges that remain for clinical translation.

### 3.1 Generating non-immunogenic cells

The main obstacle for allogeneic Treg therapy is immune rejection, driven by recognition of mismatched HLA molecules by recipient CD8^+^ and CD4^+^ T cells, as well as by NK cell surveillance. To address this, strategies have been developed to design “hypoimmunogenic” cells that can persist across HLA barriers. A cornerstone of this approach is the silencing of MHC molecules, which reduces recognition by host T cells.

#### 3.1.1 MHC silencing

The development of non-immunogenic cells for allogeneic therapies has been a focus of research in recent years. This field has evolved rapidly, driven by advances in genetic engineering techniques. The foundational work by Zijlstra et al. demonstrated the critical role of β2-microglobulin (B2M), a protein that constitutes the light chain of MHC-I, in the assembly and surface expression of MHC-I by generating B2M-deficient mice and showing that B2M-deficient cells are poorly recognized and lysed by alloreactive cytotoxic T lymphocytes (105). Building on this knowledge, researchers began exploring strategies to generate non-immunogenic cells.

Initial approaches utilized lentiviral delivery of shRNA targeting *B2M* or *HLA-A*, effectively reducing HLA class I expression in human cell lines and thereby reducing CD8^+^ T cell alloreactive responses (106). However, incomplete silencing of HLA class I expression necessitated more efficient genetic tools. More precise editing followed with the application of zinc finger nucleases by Torikai et al., enabling the knockout (KO) of the *HLA-A* locus and demonstrating the feasibility of generating HLA-A-deficient primary T cells, CAR-T cells, and embryonic stem cells capable of escaping HLA-restricted CTL-mediated lysis (107).

With the emergence of CRISPR/Cas9, a powerful genome editing tool capable of targeting multiple sites simultaneously with high efficiency and specificity, Ren et al. generated CAR T cells with triple disruption of *B2M*, *TCR*, and *PD-1* (108). Although MHC-I^−^ cells showed reduced allogeneic T cell surveillance, complete immune evasion was not achieved due to strong NK cell activation. Moreover, activated T cells still expressed MHC-II, which could accelerate rejection by allogeneic CD4^+^ T cells, highlighting the need for additional layers of editing.

To further enhance immune evasion, recent efforts have focused on targeting *CIITA*, the master regulator of MHC-II expression. Kagoya et al. generated triple-knockout (tKO) T cells by simultaneously disrupting *B2M*, *CIITA*, and *TRAC* using CRISPR/Cas9. These tKO T cells demonstrated *in vivo* persistence when exposed to allogeneic PBMCs in immunodeficient mice. When transduced with a CD19 specific-CAR, the tKO CAR-T cells persisted better than double-knockout (DKO) (*B2M* and *TRAC*) CAR-T cells in the presence of allogeneic PBMCs (109).

Alternatively, Lee et al. generated HLA class I/II-deficient T cells by targeting *B2M* and the HLA class II α chains (*DPA*, *DQA*, and *DRA*), a strategy that may avoid off-target effects on other CIITA-regulated genes. The resulting cells demonstrated reduced allogeneic T cell responses and inflammatory cytokine production while maintaining T cell functionality (110).

The emergence of iPSCs has opened a new avenue in regenerative medicine and cell therapy. Recent studies have explored the development of hypoimmunogenic iPSCs to overcome immune rejection in allogeneic transplantation. A series of articles have demonstrated the dual knocking out of *B2M* and *CIITA* across iPSCs sources efficiently abolishes MHC-I and MHC-II expression (111–115).

For Treg therapy, the rationale for MHC silencing is particularly strong, since long-term persistence in an allogeneic host is required to sustain tolerance. Eliminating MHC-I prevents host CD8^+^ T cell recognition, while MHC-II deletion prevents activation of alloreactive CD4^+^ T cells. However, complete loss of MHC molecules may also compromise physiological interactions with antigen-presenting cells and remove survival signals, raising concerns about functional fitness, infection risk, and tumor surveillance. In addition, multiplex genome editing carries the potential for off-target events or chromosomal instability, which must be carefully monitored. Thus, rigorous genomic, transcriptomic, and karyotypic quality control will be essential before clinical deployment.

Altogether, MHC silencing, whether in primary T cells or iPSCs, represents a foundational step toward generating non-immunogenic Tregs. Nevertheless, the loss of MHC molecules creates vulnerability to NK cell killing, making combinatorial strategies (i.e. co-expression of HLA-E, HLA-G, or CD47) an essential complement to MHC editing for durable immune evasion.

#### 3.1.2 Protection molecules

Silencing MHC-I expression allows cells to evade T cell-mediated immune responses, but this strategy creates a major vulnerability and is insufficient for complete immune evasion due to NK-cell mediated cytotoxicity. To address this challenge, strategies have explored the use of protective molecules that deliver inhibitory signals to NK cells, macrophages, and T cells, thereby complementing MHC editing and enabling long-term persistence of engineered cells.

Among these, HLA-E, a non-classical MHC-I molecule with limited polymorphism, acts as a key ligand for NK cell receptors, particularly the inhibitory CD94/NKG2A complex, providing an inhibitory signal that protects healthy cells from NK cell-mediated lysis (116). Using AAV-mediated gene editing, Gornalusse et al. knocked in (KI) *HLA-E* genes at one *B2M* locus in embryonic stem cells (ES), while knocking out the other *B2M* allele with a floxed HyTK fusion gene (117). Both HLA-E constructions, single-chain dimers and trimers (with peptide antigen) demonstrated the ability to prevent NK-cell-mediated lysis of ES-derived CD45^+^ cells and avoid allogeneic recognition by CD8^+^ T lymphocytes. However, this strategy is limited to NK cell subsets expressing NKG2A.

The use of HLA-G, another non-classical MHC-I molecule involved in maternal-fetal tolerance, can act via ILT2 (immunoglobulin-like transcript 2) and ILT4 (immunoglobulin-like transcript 4) inhibitory receptors on NK cells, and possibly on myeloid cells (118, 119). Its overexpression in hypoimmunogenic human pluripotent stem cells (hPSCs) effectively reduces the activity and cytotoxicity of NK cells when they were differentiated into vascular smooth muscle cells (VSMCs) (120). Co-expression of HLA-G and HLA-E may therefore provide synergistic protection, as they target distinct inhibitory pathways and together cover a broader spectrum of NK cell subpopulations.

CD47, a “don’t eat me” signal, is a ubiquitously expressed transmembrane glycoprotein, initially described for its ability to interact with Signal Regulatory Protein Alpha (SIRPα) on macrophages, thereby inhibiting phagocytosis and allowing CD47^+^ cells to evade immune clearance (121). CD47 overexpression decreases macrophage engulfment of engineered hPSC-derived VSMCs (120). Interestingly, CD47 overexpression in DKO *B2M CIITA* iPSCs, also allowed cells in undifferentiated or differentiated state to resist against NK-cell mediated killing *in vitro* and evade immune clearance when transplanted into fully allogeneic immunocompetent mice (111). Further research revealed that this protection is mediated through the SIRPα-CD47 axis, which can function as an immune checkpoint in NK cells (122). NK cell SIRPα is upregulated upon IL-2 stimulation and other environmental factors. The elevated expression of CD47 protects MHC-I-deficient targets against SIRPα^+^ NK cells. Notably, even basal CD47 expression in DKO *B2M CIITA* iPSCs and their hepatic derivatives was sufficient to prevent NK cell-mediated lysis, despite the absence of engineered overexpression (113).

Finally, Programmed Death-Ligand 1 (PD-L1) is a key immune checkpoint molecule that plays a crucial role in modulating immune responses by binding to its receptor PD-1 on T cells, thereby inhibiting their activation and cytotoxicity (123). In the context of hypoimmunogenic cell design, PD-L1 can enhance immune evasion by inhibiting CD8^+^ T cell alloreactivity (120).

Together, these strategies illustrate how layering protective molecules can compensate for the vulnerabilities created by MHC loss. For Treg therapies in particular, where long-term persistence and stable function are critical to sustain tolerance, combining MHC silencing with HLA-E, HLA-G, CD47, and/or PD-L1 expression may provide durable protection across both adaptive and innate immune barriers.

Despite these promising advances, these approaches also raise several safety considerations. First, multiplex genome editing using CRISPR/Cas9 or other nucleases carries a risk of off-target mutations and chromosomal rearrangements, which may alter genomic stability or gene regulatory networks (124, 125). Second, the disruption of MHC molecules and immune-checkpoint modulation could impair physiological immune surveillance mechanisms, potentially allowing malignant or infected cells to escape detection. Third, overexpression of inhibitory ligands such as PD-L1 or CD47, while protective against immune attack, has been associated with tumor immune evasion and may promote local immunosuppression *in vivo* (126, 127). Therefore, comprehensive preclinical evaluation including genomic integrity assays, transcriptomic profiling, and long-term tumorigenicity studies will be essential to ensure safety of next-generation hypoimmunogenic Treg therapies. Moving forward, next-generation engineered allogeneic Tregs will likely rely on carefully balanced multi-layered engineering approaches, balancing persistence, safety, and regulatory potency *in vivo*.

While gene editing and protective molecule strategies enable the generation of non-immunogenic primary Tregs, their scalability and consistency remain limited by donor availability. To overcome these constraints, attention has turned to iPSCs as a renewable and versatile source for producing standardized, hypoimmunogenic Treg products.

### 3.2 iPSCs for Treg therapy

iPSCs offer a unique opportunity to overcome the limitations of primary Treg sources by providing a renewable, scalable, and genetically malleable platform for generating standardized Treg products. Unlike donor-derived Tregs, iPSCs can be expanded indefinitely *in vitro*, differentiated into Tregs with defined phenotypes, and engineered to reduce immunogenicity or enhance function. This flexibility makes iPSCs particularly attractive for developing off-the-shelf Treg therapies, where large batches of cells with consistent quality are required. Moreover, iPSCs can incorporate the genetic modifications described above, such as MHC silencing and protective molecule expression, at the pluripotent stage, thereby producing Tregs that are both immune-evasive and functionally stable once differentiated. In this section, we review current strategies for deriving Tregs from iPSCs, recent advances in improving their regulatory phenotype, and the challenges that remain for clinical translation.

#### 3.2.1 Hematopoietic differentiation of iPSCs

The multipotency of stem cells enables their stepwise differentiation into hematopoietic progenitor cells (HPCs), which represent the gateway to generating T cells and ultimately Tregs, through protocols that recapitulate embryonic hematopoiesis. Early approaches, such as spontaneous differentiation on OP9 feeder layers, a cell line derived from mouse bone marrow stromal cells, generated limited CD45^+^ hematopoietic progenitors populations, highlighting the need for optimized signaling pathways to recapitulate embryonic development more precisely (128, 129). Mesoderm induction is initiated by bone morphogenetic protein 4 (BMP4) and Wnt signaling, which drive the expression of kinase insert domain receptor (KDR, also known as vascular endothelial growth factor receptor 2, VEGFR2), a hallmark marker of early mesoderm and hemangioblast specification (130). Subsequent hematopoietic specification relies on vascular endothelial growth factor (VEGF) and fibroblast growth factor 2 (FGF2), which guide mesodermal cells toward hemogenic endothelium, further sustained by cytokines like stem cell factor (SCF), fms-like tyrosine kinase 3 ligand (FLT3L), and interleukin-3 (IL-3) to support endothelial-to-hematopoietic transition (131). This coordinated signaling cascade ultimately yields CD34^+^ progenitors, with CD45 and CD43 serving as lineage-specific markers for lymphoid/myeloid commitment and hematopoietic maturation, respectively.

Two primary strategies dominate HPCs generation: 2D monolayer cultures and 3D embryoid body (EB) systems. 2D monolayer cultures on defined matrices offer reproducibility and scalability, producing CD34^+^CD43^+^CD45^+^ progenitors in chemically controlled conditions suitable for downstream clinical application (132, 133). Conversely, EB-based methods aggregate pluripotent cells into 3D spheroids, mimicking embryonic spatial organization to generate similar progenitors (134, 135). While EBs better replicate developmental complexity, their heterogeneity in size and differentiation gradients poses standardization challenges when adapting protocols to GMP-grade production.

For the purpose of Treg therapy, the generation of robust HPCs is a critical prerequisite, as these progenitors must then undergo Notch-driven thymic differentiation to yield stable FOXP3^+^ regulatory T cells. Ensuring high-quality, lymphoid-competent HPCs at this stage is therefore essential for the efficiency and reproducibility of downstream Treg differentiation.

#### 3.2.2 T cell differentiation

##### 3.2.2.1 Notch signaling

Notch signaling is indispensable for T-cell lineage commitment and orchestrates critical checkpoints from early thymic entry onward. In the thymus, Notch1 activation drives multipotent hematopoietic progenitors toward the T-cell lineage while suppressing alternative fates such as B-cell, myeloid, or NK cell differentiation (136–138). The process is mediated by thymic epithelial cells (TECs) expressing Delta-like ligand 4 (DLL4), the dominant and most potent Notch ligand engaging Notch1 receptors on progenitor cells to initiate T-cell specification (139, 140). Delta-like ligand 1 (DLL1) also contributes to T-cell lineage commitment, though with lower efficiency (141). The Notch ligands Jagged1/2 are not essential for canonical αβ T-cell lineage commitment, although Jagged2 deficiency in mice disrupts thymic development and influences γδ T/NK cell differentiation (142, 143).

Maintenance of T-cell commitment requires sustained Notch1-DLL4 interactions during early thymic stages, preventing lineage diversion and promoting survival together with interleukin-7 receptor (IL-7R) signaling (144). Notch1 is also necessary for the transition from the double-negative (DN) to the double-positive (DP) stage (145). Conditional inactivation of Notch1 at DN2-DN3 impairs Vβ-DJβ recombination efficiency, causing a developmental block at DN3 due to defective TCRβ rearrangement (146). Notch signaling further cooperates with pre-TCR signals at the β-selection checkpoint to ensure metabolic fitness and survival of thymocytes expressing functional TCRβ chains, eliminating those with nonproductive rearrangements (147, 148).

For iPSC-derived Treg therapies, robust Notch activation is a non-negotiable requirement to drive hematopoietic progenitors into the T-cell lineage. Practically, this is achieved using DLL4-based systems, including stromal co-cultures, DLL4-coated surfaces, and artificial thymic organoids (ATOs) that mimic thymic Notch engagement. However, while Notch is essential for generating conventional T-cell precursors, it does not specify the Treg lineage. Additional cues, including TCR signal strength and cytokines such as IL-2 and TGF-β, are required to stabilize FOXP3 expression and commit developing thymocytes to a regulatory fate. Thus, Notch provides the essential gateway signal in iPSC-derived Treg generation, laying the foundation for subsequent lineage-defining signals.

Once iPSC-derived hematopoietic progenitors are specified toward the T-cell lineage via Notch signaling, appropriate culture systems are required to support their full maturation. Several strategies have been developed, each with distinct advantages and limitations.

##### 3.2.2.2 2D culture: OP9 stromal cells expressing Notch ligands

The OP9-DLL1 co-culture system has been a cornerstone for studying T cell development and generating T cells from various hematopoietic sources. Developed by Schmitt and Zúñiga-Pflücker, the system uses OP9 stomal cells engineered to express DLL1 or DLL4 (149). This system efficiently supports T cell differentiation from mouse hematopoietic stem cells and embryonic stem cells (150, 151). The OP9-DLL1 system was later extended to human HSCs, ESCs, and iPSCs (152, 153). However, OP9 cocultures typically struggles to generate mature single-positive (SP) CD8^+^ and CD4^+^ T cells, often resulting in an accumulation of immature CD4^+^CD8^+^ DP precursors and rare naïve CD8^+^ SP T cells (152, 154). For Treg applications, this immaturity and stromal dependence significantly limit translational potential.

##### 3.2.2.3 3D culture: artificial thymic organoid – MS5-DLL1/DLL4

To better mimic physiological thymic architecture, 3D ATOs were developed. These consist of MS5 stromal cells engineered to express Notch ligand DLL1/DLL4 and aggregated with hematopoietic progenitor. Early studies demonstrated the ability of these structure to better mimic physiological thymic niches, allowing efficient and reproducible *in vitro* differentiation of human hematopoietic stem and progenitor cells into mature and functional CD8^+^ SP and CD4^+^ SP T cells with a diverse TCR repertoire (155). The model has since been applied to iPSCs, pluripotent cells are directed towards mesoderm and then aggregated into the ATO system. After a hematopoietic induction followed by a T cell differentiation phase, mature CD8^+^ and CD4^+^ T cells are successfully generated from iPSCs (156). Due to a better yield obtained with MS5 expressing the Notch ligand DLL4 compared to the MS5-DLL1 system, subsequent research has converged towards the use of DLL4 ligand. This system was then applied to genetically modified iPSCs expressing CAR to generate CAR-T cells (157). Importantly, the microenvironment of ATOs provide a platform that may in principle support positive and negative selection events needed to generate functional FOXP3^+^ Tregs, although this remains an active area of investigation.

##### 3.2.2.4 Feeder-free differentiation system

To overcome the translational limitations of stromal cell-dependent co-cultures, several feeder-free Notch-based systems have been developed. Iriguchi et al. established a feeder-free protocol using DLL4-coated plates combined with defined cytokine cocktails including SCF, FLT3L, IL-7, thrombopoietin (TPO), and stromal cell-derived factor 1 alpha (SDF1α). While this approach successfully generated mature CD8^+^ SP T cells from T-cell-derived iPSCs (T-iPSCs) and TCRs-transduced iPSCs, this system poorly performed for iPSCs lacking pre-existing TCR rearrangements (158). By using DLL4-conjugated microbeads (DLL4-µbeads), Trotman-Grant et al. enabled the generation of CD4^+^, CD8^+^ DP cells expressing CD3 and TCR*αβ* from fibroblast-derived iPSCs (159). Building on this, more recent studies have shown that combining vascular cell adhesion molecule-1(VCAM1) with DLL4 during the endothelial-to-hematopoietic transition (EHT) synergistically enhances Notch signaling and markedly improved the yield of T cell-competent progenitors, enabling efficient generation of mature CD8αβ^+^CD3^+^TCRαβ^+^ T cells (160). Recently, a commercially available research-grade kit (STEMdiff™ T Cell Kit, Stem cell) has been developed to support the entire differentiation process from human iPSCs to mature CD8^+^ T cells in a feeder-free and serum-free setting, although details of its formulation are proprietary.

Overall, feeder-free systems efficiently generate mature CD8^+^ T cells but face challenges in producing functional CD4^+^ T cell subsets, restricting their use to applications where CD8-biased differentiation is advantageous, such as CD8^+^ Treg development.

##### 3.2.2.5 Comparative perspective

Each culture platform presents distinct advantages and limitations: OP9 cultures are simple but stall at DP stage and rely on murine stromal cells; ATOs best recapitulate thymic selection and generate CD4^+^ and CD8^+^ SP T cells with a diverse TCR repertoire; feeder-free systems are scalable and GMP-compatible, with a strong CD8^+^ bias, but limited CD4^+^ output. From the perspective of developing iPSC-derived CD8^+^ Treg therapies, this intrinsic CD8 skew is advantageous, although additional cues are required to enforce regulatory fate. In particular, incorporating cytokines such as IL-2 and IL-15, along with modulators like rapamycin, may help stabilize the FOXP3^+^ phenotype and enhance suppressive function. DLL4-based ATOs remain attractive for recapitulating thymic-like selection processes to Treg commitment, whereas feeder-free platforms may offer the most practical route for large-scale CD8^+^ Treg manufacturing if paired with strategies that bias differentiation toward a stable regulatory lineage.

Beyond differentiation efficiency, functional maturation represents a major remaining challenge. Several systems induce premature TCR expression, diverging from canonical thymic ontogeny and potentially altering selection thresholds or lineage commitment, an issue noted in both foundational developmental studies and recent comparative analyses. This ontogenetic mismatch raises a broader question for iPSC-derived Tregs: whether they should rely on their endogenous TCR repertoire (with differences between fibroblast-derived iPSCs, T-iPSCs, polyclonal vs monoclonal repertoires) or instead be equipped with a CAR providing defined specificity and more predictable functionality. CARs may help overcome selection defects and confer targeted action (161), whereas endogenous repertoires offer physiological recognition but introduce variability and safety considerations.

A related bottleneck is the limited functional characterization reported in many studies. Comprehensive assessments of suppressive function, cytokine secretion, metabolic fitness, stability under inflammatory stress, and antigen-specific versus antigen-independent mechanisms are often incomplete. This is particularly important for CD8^+^ Tregs, whose suppressive pathways, including IL-34 production, cytolytic mediators, and bystander effects, may not strictly depend on antigen specificity, making them potentially compatible with either endogenous TCRs or CAR-based targeting. Existing regulatory CAR designs (i.e. anti–HLA-A2, anti–BCR, alloantigen-specific CARs) illustrate the range of possibilities, but their optimal configuration for iPSC-derived CD8^+^ Tregs remains to be defined.

Altogether, current iPSC-based protocols make it feasible to generate mature CD8^+^ T cells at scale, providing a solid foundation for CD8^+^ Treg development. The central challenge now lies in ensuring stable regulatory identity and robust suppressive function, which will require coupling differentiation platforms with precise regulatory programming and stringent functional validation.

## 4 Switch T cells into Treg phenotype

### 4.1 Reprogram Tconv into Treg

Beyond isolating natural Tregs or deriving them from iPSCs, an alternative approach is to convert conventional T cells (Tconv) into regulatory cells. This strategy leverages the abundance and accessibility of Tconv as starting material, aiming to reprogram them toward a stable suppressive phenotype. Different methods have been explored, including direct genetic reinforcement of lineage-defining transcription factors and the use of optimized culture conditions to impose a regulatory program. Both approaches have shown potential, though each faces challenges in terms of long-term stability and functional fidelity, particularly under inflammatory conditions.

#### 4.1.1 FOXP3 overexpression

FOXP3 is the master transcription factor that defines Treg identity. Tconv can be reprogrammed into Tregs through overexpression of FOXP3. Initial attempts to generate suppressor T cells from Tconv via retroviral-mediated FOXP3 overexpression proved insufficient, as the resulting cells lacked stable suppressive function *in vitro* (162). However, subsequent work employing lentiviral vectors demonstrated improved outcomes, with FOXP3-transduced CD4^+^ Tconv acquiring stable suppressive activity and inhibiting effector T-cell proliferation *in vitro* (163). More recently, increased FOXP3 expression via retroviral vectors in CD4^+^ Treg themselves stabilized the regulatory phenotype, preventing reversion to effector identity under inflammatory conditions (164). In CD8^+^ T cells, FOXP3 overexpression can modulate metabolism (165) and its ability to confer full suppressive activity remains uncertain, but evidence suggests that additional transcriptional regulators must cooperate with FOXP3 to fully establish a stable CD8^+^ Treg program (92).

However, FOXP3 expression alone is often insufficient to ensure durable Treg lineage commitment. Stable regulatory T cell identity requires consolidation of the transcriptional and epigenetic program that maintains suppressive function. In particular, transient FOXP3 expression can occur in activated conventional T cells, which may subsequently lose suppressive properties and revert to effector phenotypes under inflammatory conditions. Stable Tregs are therefore characterized by epigenetic fixation of the regulatory program, most notably through demethylation of the FOXP3 Treg-specific demethylated region (TSDR), which correlates with sustained FOXP3 expression and lineage commitment. Resistance to inflammatory reprogramming, maintenance of suppressive activity, and preservation of the regulatory transcriptional network are critical parameters when evaluating engineered or induced Treg populations. In the context of cell therapy, minimal criteria for a stable Treg product typically include sustained FOXP3 expression, TSDR demethylation, robust suppressive function *in vitro*, and phenotypic stability after exposure to pro-inflammatory signals.

#### 4.1.2 Optimized medium

In addition to providing optimal culture conditions for maintaining the Treg phenotype in an autologous strategy, these same conditions are sufficient to promote the conversion of Tconv into Tregs. TGF-β has been shown to initiate FOXP3 expression in CD4^+^CD25^-^ T cells under TCR stimulation (166, 167), yet the resulting induced CD4^+^ Tregs (iTregs) often exhibit unstable FOXP3 expression and limited suppressive function (168, 169). IL-2 has been demonstrated as indispensable for TGF-β-mediated FOXP3 induction in CD4^+^ Tregs, stabilizing its expression and enabling robust proliferation of functional iTregs with potent immunosuppressive activity (170, 171), and to support expansion and function of CD8^+^CD103^+^ Tregs (172) but not CD8^+^CD45RC^low/–^ Tregs, reflecting subset-specific cytokine dependencies. Rapamycin, an mTOR inhibitor, further enhances TGF-β-dependent FOXP3 expression in human naive CD4^+^ T cells and induces suppressor function *in vitro* and *in vivo*, as evidenced in preclinical GvHD models (173). In combination with ATRA, rapamycin augments the expression of gut-homing markers, thereby improving both functionality and tissue-targeting of Tregs (30, 32, 33). Combining these signals with robust TCR stimulation (via high α-CD3/CD28 bead ratios) is essential for generating stable human iTregs with sustained immunosuppressive capacity, as highlighted by Kim et al. (174).

However, despite these advances, challenges remain in maintaining iTregs long-term stability by preventing reversion to effector phenotypes under inflammatory conditions. This limitation has motivated complementary strategies to derive Tregs directly from iPSCs, which offer greater scalability and genetic flexibility.

### 4.2 IPSC-directed differentiation toward Tregs

The use of iPSCs provides a renewable and genetically malleable source for Treg generation. For more than a decade, the study by Haque et al. stood as the sole reference demonstrating generation of functional Tregs from iPSCs. In this work, mouse iPSCs were retrovirally transduced with FOXP3 and co-cultured on OP9-DLL1 stromal cells for 30 days, yielding CD4^+^CD25^+^FOXP3^+^ Tregs alongside a minor population of CD8^+^ Tregs (175). These cells secreted immunosuppressive cytokines IL-10 and TGF-β, suppressed effector T-cell proliferation *in vitro*, and reduced arthritis incidence and clinical scores in mouse models upon adoptive transfer (175).

More recently, Yano et al. reported the first successful generation of human iPSC-derived CD4^+^ regulatory T-like cells. Their protocol combined embryoid body-based differentiation with aggregation in ATO containing MS5-DLL4 stromal cells, followed by feeder-free expansion and FOXP3 induction using a cocktail of small molecules and cytokines (AS2863619, MR2-1, rapamycin, and TGF-β) (176). The resulting iPSC-derived CD4^+^ Treg-like cells exhibited natural FOXP3 expression, demonstrated potent *in vitro* suppressive functions, and, when engineered with CARs for antigen specificity, improved survival in murine GvHD models (176), highlighting a key strategic choice between endogenous TCR versus CAR-equipped iPSC-Tregs. To address scalability and regulatory limitations of ATO systems, Fong et al. developed an alternative workflow using the STEMdiff™ T Cell Kit to efficiently generate DP cells from iPSCs, followed by PMA/ionomycin stimulation to obtain CD4^+^ SP T cells, and the Immunocult Human Treg Differentiation Supplement (StemCell Technologies) to confer suppressive activity (177).

iPSC-derived Tregs offer an exciting scalable platform for allogeneic therapy, although their full translational readiness warrants distinction from current proof-of-concept achievements. Notably, while thymic Tregs acquire stable epigenetic identity through active TSDR demethylation at the FOXP3 locus during thymic selection (178). Recent protocols, including those reported by Yano et al. and Fong et al. demonstrate comparable levels of FOXP3 demethylation to primary Tregs.

However, these *in vitro* differentiation approaches cannot fully recapitulate the thymic microenvironment, including stromal interactions and AutoImmune Regulator (AIRE)-mediated selection, raising questions about long-term epigenetic fidelity. Critically, stability of TSDR demethylation of this new generation of iPSC-derived Tregs under *in vivo* inflammatory stress remains unproven, potentially limiting persistence and risking conversion to effector phenotypes.

Despite these advances, current iPSC-derived Treg protocols remain largely restricted to the CD4^+^ lineage, with no robust method yet available for producing stable and functional CD8^+^ Tregs from iPSCs. Furthermore, reliance on ATOs, particularly on mouse stromal cells and complex 3D organoid culture systems, poses logistical and regulatory barriers to GMP adaptation and large-scale manufacturing. Ongoing efforts aim to optimize feeder-free, scalable platforms and incorporate lineage-stabilizing cues to fully unlock the therapeutic potential of iPSC-derived Tregs as standardized, off-the-shelf products.

### 4.3 Comparative biological and translational features of CD4^+^ and CD8^+^ Tregs

While CD4^+^ Tregs have historically been the primary focus of regulatory T-cell therapies, increasing evidence supports the therapeutic potential of CD8^+^ Tregs. These two regulatory lineages share several core features, including FOXP3-dependent regulatory programs and the ability to suppress effector immune responses, but they also exhibit important biological and translational differences.

CD4^+^ Tregs primarily recognize antigens presented by MHC class II molecules and exert immunoregulation through multiple mechanisms, including cytokine secretion, metabolic disruption, and modulation of antigen-presenting cells. Their biology and therapeutic potential are supported by extensive preclinical and clinical data, and they currently represent the most advanced platform in clinical development.

In contrast, CD8^+^ Tregs recognize antigens presented by MHC class I molecules, enabling them to interact directly with a broader range of nucleated cells, including parenchymal cells within target tissues. Several studies suggest that CD8^+^ Tregs exhibit potent suppressive activity and durable regulatory function, potentially enabling more localized and antigen-specific immune control. In addition, emerging data indicate that CD8^+^ Tregs may display distinct metabolic and transcriptional programs that contribute to their stability and functional specialization.

From a translational perspective, CD4^+^ Tregs currently benefit from a more mature clinical evidence base and established manufacturing workflows. By contrast, CD8^+^ Tregs remain at an earlier stage of development but may offer unique advantages related to tissue-level immune regulation and antigen-specific targeting. However, CD4^+^ Tregs remain the only regulatory subset with substantial clinical validation to date, and the potential advantages of CD8^+^ Tregs, including enhanced durability or tissue-level immune control, remain hypotheses that require further confirmation in human therapeutic settings.

Together, these complementary features suggest that CD4^+^ and CD8^+^ Tregs should not necessarily be viewed as competing therapeutic platforms but rather as potentially synergistic regulatory populations that may be harnessed in different clinical contexts.

## 5 Autologous therapy vs iPSC-derived Tregs therapy

Autologous Treg therapy has paved the way for clinical translation, with multiple phase I/II trials establishing its feasibility and safety in transplantation and autoimmunity (179). However, while highly personalized and generally well tolerated, this individualized strategy presents several limitations that can hinder its clinical applicability and scalability (**Table 6**).

### 5.1 Insights from autologous Treg clinical trials

#### 5.1.1 Transplantation

Autologous Treg-based therapies have shown promising results in various transplantation contexts, particularly for the management of GvHD and solid organ transplant (SOT) rejection.

In early pioneering work, a single infusion of CD4^+^CD25^high^CD127^-^ Tregs successfully alleviated chronic GvHD symptoms affecting the skin, liver, and lungs in a patient infused with a low dose (1 × 10^5^ cells/kg) and led to a temporary, yet longer-lasting improvement in symptoms compared to conventional IS in a patient with acute GvHD infused with 3 × 10^6^ cells/kg (34) (**Table 3**). In a subsequent study involving 34 patients with hematologic malignancies undergoing HLA-matched umbilical cord blood stem cell transplantation, infusion of up to 3 × 10^8^/kg *ex vivo*-expanded CD4^+^CD25^+^FOXP3^+^ Tregs from cord blood was found to be safe and well-tolerated, with only mild (grade 1–2) post-infusion adverse events (11, 54). A grade 3 toxicity (transient hypertension in two patients) was observed but managed effectively using standard clinical care, with no evidence of increased risk for relapse, chronic GvHD, or reduced survival. Furthermore, the incidence of opportunistic infections in Treg-treated patients was comparable to those receiving standard immunosuppression (IS). To maximize the interpretability of these safety outcomes, donor–recipient matching is critical and, whenever possible, was rigorously applied to ensure reliable conclusions on safety and efficacy despite small cohort sizes.

The feasibility and safety of Treg infusion have likewise been demonstrated in recipients of kidney, liver, heart or islet transplants. In phase I clinical trials, administration of CD4^+^CD25^+^CD127^-^ Tregs in kidney transplanted patients was not associated with infusion reactions or serious treatment-related adverse events, including cytokine release syndrome, infectious complications, malignancies, or clinical rejection for up to two years post-infusion (“TASKp” and “TRACT” trials). A transient grade 3 leukopenia potentially related to Treg infusion was observed in only one patient, and spontaneously resolved (19). Two patients developed donor-specific antibodies, though these were likely due to nonadherence to immunosuppression or intolerance rather than the cell therapy itself (31).

Further support for the safety of Treg-based therapies comes from the ONE Study consortium, a major EU-funded initiative that evaluated the safety of six cell therapy products, including polyclonal and donor-reactive Tregs, in seven single-arm phase I/IIa trials of kidney transplant recipients from living donors (66, 69, 180, 181). Patients received a single cell infusion from 5 x 10^5^ to 1 x 10^7^ cells/kg (except darTreg, 2 x 10^3^ to 2 x 10^6^ cells/kg) 7 days before to 10 days after transplantation instead of basiliximab, with optional MMF withdrawal at 1 year. Approximately 40% of Treg-treated patients were successfully tapered to tacrolimus monotherapy and returned to immune homeostasis, with similar incidence and timing of acute rejection episodes as in IS-treated patients. They did not experience higher rates of adverse events and, remarkably, had statistically lower incidence of viral infections (66). A downstream follow-up study, the TWO Study (Transplantation Without Over-Immunosuppression), is currently underway to evaluate the efficacy of this cell therapy dose in renal transplant recipients. Only preliminary results from 7 patients treated under the original protocol have been published, showing 100% survival and no acute rejection at 18 months in the Treg-treated arm (182). Building on the experience of the ONE and TWO Studies, the EU-funded ReSHAPE consortium (*Reshaping undesired Inflammation in challenged Tissue Homeostasis by Next-Generation Treg Approaches*, *https://www.reshape-h2020.eu*) was established to advance regulatory T cell therapy beyond first-generation autologous Tregs. Within this framework, several first-in-human trials are planned following a 3 + 3 dose-escalation design. Our lab contributes directly to this initiative by launching the first autologous CD8^+^ Treg clinical trial in solid organ transplantation, the “Eight-Treg” study (NCT06777719), a milestone that will test the feasibility, safety, and immunoregulatory potential of CD8^+^ Tregs in humans. This effort highlights both the translational ambition of ReSHAPE and the field’s shift toward novel regulatory subsets and universalized manufacturing strategies.

Four clinical trials have reported the safety of CD4^+^ Treg infusion in liver transplant recipients, three of which were phase I/II studies demonstrating reduced immunosuppression requirements and decreased anti-donor responses (75–77) (ARTEMIS, NCT02474199; ThRIL, NCT02166177; UMIN-000015789). Two additional trials are currently ongoing (LITTMUS-MGH, NCT03577431; LIBERATE, NCT05234190).

In the field of islet transplantation, one trial established proof-of-concept for safety using human islet donors (82) (NCT04820270), while a pilot xenotransplantation study has opened new perspectives (80) (NCT03162237).

When starting from freshly collected blood cells, determining the optimal time for infusion can be challenging. Most trials have focused on GVHD prophylaxis, with Tregs administered within the first days or weeks following transplantation (**Table 3**). Similarly, in SOT, trials generally aim to infuse cells during the perioperative period, though recruitment windows extend up to one year for feasibility reasons (**Table 4**) (76).

In Hematopoietic Stem Cell Transplantation (HSCT), Tregs are manufactured from the bone marrow (BM) or peripheral blood (PB) of the transplant donor, and from a third-party umbilical cord blood (UCB) donor in UCB transplantation. In solid organ transplant (SOT), Tregs are generally derived from the recipient’s blood, with the exception of one trial using thymus-derived Tregs, which demonstrated safety in heart transplant patients (78, 79) (NCT04924491).

Genetically modified Tregs are now entering clinical evaluation. These modifications include enzymatic fucosylation (NCT02423915), lentiviral IL-10 expression (NCT06462365), or other undisclosed engineering strategies (ORCA, NCT06195891). Antigen specificity can be conferred through CARs (NCT05993611, NCT04817774, NCT05234190) or by ex vivo culture with allogeneic stimulator cells from SOT donors (NCT02091232, NCT02244801, UMIN-000015789, NCT01624077, NCT02188719, NCT02474199, NCT03577431, NCT03654040) or from HSCT recipients (NCT01795573, NCT04640987).

#### 5.1.2 Autoimmune diseases

In both adults and children with T1D, the administration of *ex vivo*-expanded CD4^+^CD25^+^CD127^-^ Tregs has proven to be safe and well tolerated in five clinical trials (NCT01210664, ISRCTN06128462, EudraCT: 2014-004319-35, NCT02691247, NCT02772679), and four additional trials are ongoing (**Table 2**). In adults, a single infusion of Tregs resulted in no infusion-related reactions, opportunistic infections, malignancies, or other serious adverse events. Among the 14 adult patients treated, 11 grade 3 or 4 adverse events occurred during the 31-month follow-up period, but these were largely attributed to the underlying disease itself, with no association between dose and adversed events (18, 39, 40). Similarly, in pediatric patients, infusion of CD4^+^ Tregs in 12 children recently diagnosed (less than 2 months) resulted in no episodes of acute hyperglycemia, hypoglycemia, or other adverse effects during the 4–5 months of follow-up (41, 42). Importantly, treatment was associated with reduced exogenous insulin requirements and lowered glycated hemoglobin within two weeks of infusion, suggesting a potential therapeutic benefit. In a phase II trial, single infusions of polyclonal expanded Tregs (expTregs) did not prevent β-cell decline over one year. Instead, improved C-peptide preservation correlated with Treg quality, defined by lower fold expansion and its transcriptional signature, rather than infused dose (46).

Beyond T1D, Treg therapy is now being investigated across multiple autoimmune diseases. In amyotrophic lateral sclerosis (ALS), two studies (NCT03241784; NCT04055623) demonstrated the safety of autologous Treg infusions combined with low-dose IL-2, along with signals of slowed disease progression (36); four additional trials have recently been initiated (**Table 2**). In systemic sclerosis, a phase I/IIa trial confirmed safety and tolerability of autologous Tregs and reported clinically meaningful symptomatic improvement (38). Treg infusion has also proven safe in IBD, with dose-dependent early efficacy signals (49, 51). Similarly, studies in multiple sclerosis (MS) have confirmed safety (37) and continue to evaluate therapeutic potential (NCT06566261), while functionally altered CD8^+^ Tregs in severe MS cases suggest potential for CD8^+^ Treg therapies (183). In systemic lupus erythematosus (SLE), early exploratory trials are assessing the safety and feasibility of adoptive Treg transfer, though available data remain limited and conclusive efficacy has yet to be demonstrated (53). In autoimmune hepatitis, two trials (NCT02704338, IRAS ID: 177127) have evaluated the infusion of *ex vivo*–expanded polyclonal Tregs. Early reports emphasize feasibility and safety (47, 48), while definitive efficacy data remain pending. In pemphigus vulgaris, a pilot study (NCT03239470) reported no serious adverse events following adoptive Treg transfer, supporting further evaluation of tolerance induction toward desmoglein autoantigens to reduce corticosteroid dependence. Finally, Treg-based interventions have recently begun in IPEX syndrome (NCT05241444) and rheumatoid arthritis (RA) (NCT06201416).

Most AID trials have used autologous blood-derived Tregs, with the exception of three studies employing allogeneic UCB–derived Tregs from the Zhou laboratory (Second Xiangya Hospital; NCT02932826, NCT03011021) and the Shneider laboratory (Cellenkos, Inc; NCT05695521). UCB-derived Tregs have also been shown to be safe and to improve clinical outcomes when infused in patients with non-autoimmune bone marrow disorders (84, 85), as well as in COVID-19 patients (83), for whom thymus-derived Tregs are currently under investigation (THYTECH2, NCT06052436). By contrast, a trial using allogeneic blood–derived Tregs was terminated following the pandemic (NCT04482699). Alzheimer’s disease and hidradenitis suppurativa are two non-autoimmune conditions currently under investigation for autologous Treg cell therapy (NCT03865017, NCT06361836).

These findings collectively support the concept that Treg-based therapies are logistically feasible and can be administered safely, with a consistent reduction in pharmacologic IS requirements and associated toxicities, including decreased infection rates. Most strategies rely on the infusion of CD4^+^FOXP3^+^ Tregs, though a few approaches employ Tr1 cells (49, 56, 64, 65). The majority of protocols use autologous blood or HSC donor–derived Tregs (allogeneic to the recipient but autologous to the reconstituted immune system), while alternative sources such as allogeneic UCB or autologous thymus remain limited in cell number and have so far been applied only in select cases. Despite a wide range of doses tested, these trials consistently demonstrate safety, while clear efficacy signals remain preliminary, and the durability of clinical benefit has yet to be firmly established.

#### 5.1.3 Comparative translational perspective across Treg platforms

Taken together, current Treg therapeutic strategies can be broadly divided into three platforms with distinct translational profiles: autologous products, donor-derived allogeneic products, and iPSC-derived engineered products. Autologous Tregs currently represent the most clinically mature platform, with the strongest safety dataset across transplantation and autoimmune diseases, but remain constrained by individualized manufacturing, variable starting material, and limited scalability. Donor-derived allogeneic Tregs, including umbilical cord blood- and third-party-derived products, offer improved accessibility and partial standardization, yet raise challenges related to host-versus-graft rejection, limited persistence, and donor-dependent heterogeneity. By contrast, iPSC-derived Tregs remain largely preclinical but offer the greatest potential for scalable, standardized, and extensively engineered off-the-shelf products. Their development, however, depends on overcoming major bottlenecks, including robust differentiation into stable regulatory cells, control of immunogenicity, protection from host immune rejection, and implementation of additional immune-evasion strategies when MHC molecules are edited. Across all platforms, safety assessment must extend beyond immediate infusion tolerance to include persistence, lineage stability, off-target immunosuppression, infection risk, tumor surveillance, and *in vivo* trafficking, underscoring the need for harmonized monitoring frameworks as the field moves toward increasingly engineered products.

### 5.2 What’s next?

Several phase I/IIa clinical trials involving CD4^+^ Tregs *ex vivo*-expanded from peripheral blood have demonstrated that their administration is safe, well-tolerated, and associated with early signs of therapeutic efficacy. These studies consistently reported no infusion-related toxicity, no increased risk of opportunistic infections or malignancies, and in some cases, indications of immunomodulatory benefit, particularly in settings such as graft-versus-host disease, kidney transplantation, and autoimmune diseases, providing a solid foundation for regulatory cell therapy.

#### 5.2.1 CD8^+^ Tregs therapy

The field is now entering a new phase with CD8^+^ Tregs, which offer stronger per-cell suppressive potency and complementary mechanisms of action (184). CD8^+^ T cells have been extensively used clinically as effector cells for the treatment of cancers (185, 186) and chronic infections (NIAID 2008). Tumor-infiltrating lymphocytes (TILs), primarily composed of highly cytotoxic CD8^+^ T cells, have been administered to patients with metastatic melanoma in doses ranging from 1 x 10^9^ to 2 x 10^11^ cells/kg (with a minimum threshold of 5 x 10^8^ cells/kg) and have generally been well-tolerated, with no unexpected serious adverse events (NCT01118091) (187). The EIGHT Treg study (NCT06777719) is assessing for the first-time cell therapy based on *ex vivo*-expanded CD8^+^ Tregs from peripheral blood in SOT. These cells possess a natural capacity to modulate inflammatory responses and are expected to play a crucial role in preventing renal graft rejection. The primary objective of the EIGHT Treg study is to evaluate the safety and feasibility of administering CD8^+^ Tregs in place of conventional induction immunosuppression, while maintaining a standard maintenance IS regimen for patient protection. A secondary objective is to assess efficacy through clinical endpoints and exploratory biomarkers. This represents a milestone, as CD8^+^ Tregs may provide more robust control of alloimmunity than their CD4^+^ counterparts, owing to their distinct antigen-recognition pathways and suppressive potency.

#### 5.2.2 Large-scale production, cost, universality

**Figure 1:**
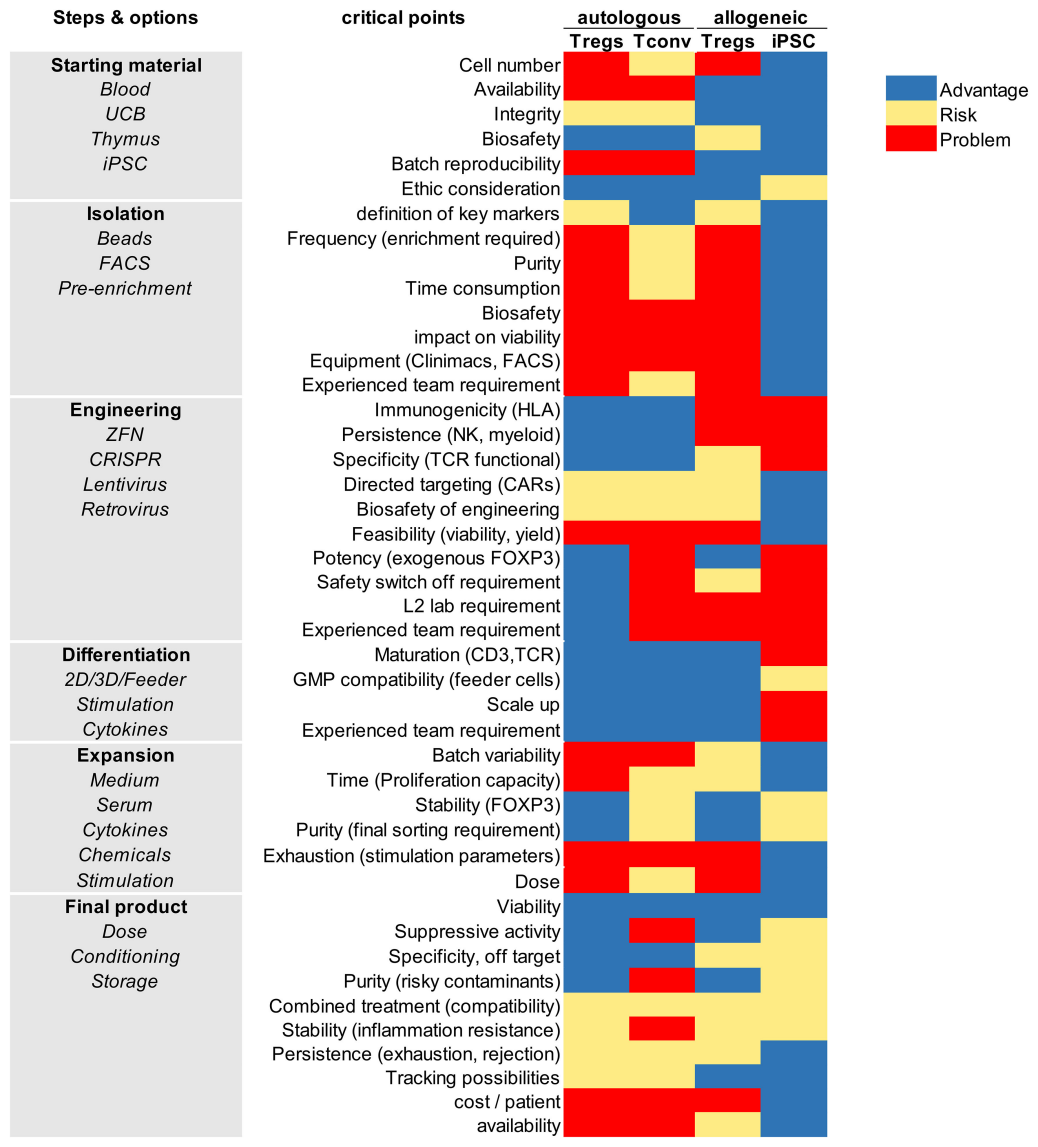
Design and manufacturing landscape of Treg therapies: comparative advantages and constraints of autologous and allogeneic approaches. Overview of the main development workflows for regulatory T cell (Treg) therapies derived from different platforms (autologous, donor-derived allogeneic, and iPSC-derived). The heatmap summarizes key translational parameters across platforms, highlighting relative advantages, limitations, and technical bottlenecks at different stages of development (cell sourcing, manufacturing, engineering, and clinical translation). Colors indicate qualitative categories (advantage, limitation, or technical challenge).

The **Figure 1** provides a visual summary of critical attributes across the manufacturing workflow of each step and therapeutic modality, with advantages shown in blue, risks in yellow, and major limitations or challenges in red.

Autologous therapies are inherently personalized, as each product must be derived from the individual patient’s cells, expanded *ex vivo*, and quality-controlled before reinfusion. This complex and time-consuming process delays treatment, restricts access to specialized facilities and generate substantial costs. Timelines are particularly problematic for patients with acute conditions requiring prompt intervention. Additionally, the quality and quantity of autologous cells may be variable and suboptimal, especially in elderly or immunocompromised patients, potentially compromising therapeutic efficacy.

In contrast, IPSC-derived cell therapy offers the possibility of generating standardized “off-the-shelf” products from healthy donors. This approach supports large-scale production of high-quality cells, reducing both manufacturing time and cost. Allogeneic products also facilitate implementation of advanced gene editing strategies to reduce immunogenicity and enhance cell functionality. However, challenges remain, including immune rejection and the need for immune-evasive modifications to ensure long-term persistence and efficacy across HLA barriers.

In this framework, iPSC-derived Tregs represent the next frontier, combining scalability, genetic flexibility, and universal applicability. These products could integrate immune-evasive modifications (MHC editing, protective molecules), lineage-stabilizing circuits (FOXP3 reinforcement, epigenetic control), and engineered specificity (CARs, TCRs) to generate standardized, potent Treg therapies (188). However, iPSC-Treg manufacturing is still in its early stages. Major challenges remain in achieving stable and efficient differentiation into CD4^+^ or CD8^+^ Tregs, maintaining FOXP3 expression under inflammatory conditions, and translating complex organoid- or stromal-based systems to fully GMP-compatible workflows.

Despite these challenges, the potential of IPSC-derived allogeneic cell therapy to overcome key limitations of autologous approaches, and to deliver universal, rapidly available and functionally enhanced Treg products, positions it as a compelling strategy for broader clinical application. Beyond transplantation, such universal Treg therapies may benefit autoimmune, inflammatory or fibrotic diseases in which fast, standardized, and potent immunoregulation is required.

## 6 Perspectives and outlook

Regulatory T cell therapy has progressed from proof-of-concept studies to early-phase clinical trials in transplantation and autoimmune diseases, consistently demonstrating feasibility and safety. As the field moves toward establishing clinical efficacy, several scientific and technical parameters are emerging as critical determinants of success. Regulatory T cells are unlikely to function as stand-alone interventions and are most often administered in combination with conventional immunosuppressive regimens. Careful consideration of drug-cell interactions is therefore required, as commonly used agents can differentially affect regulatory T cell survival and function.

Adjunctive strategies aimed at enhancing Treg persistence and specificity are being actively explored. These include the use of low-dose interleukin-2 to selectively support regulatory populations (39), engagement of checkpoint pathways to sustain suppressive activity, and antigen-specific approaches such as peptide-based targeting strategies or engineered receptors targeting transplant mismatches (NCT05234190, NCT04817774, NCT06566261), inflamed organ (Eudract 2006-004712-44, NCT02327221), CD6 (NCT05993611) or autoimmune antigens such as citrullinated protein (NCT06201416, NCT06361836). While these interventions hold promise, their integration into clinical protocols requires rigorous evaluation to balance efficacy, safety, and durability of immune regulation.

Emerging analytical and computational approaches may also contribute to the optimization of Treg therapies. Integration of high-dimensional immune profiling with clinical and histological data has the potential to refine patient stratification, identify biomarkers of stability and function, and inform longitudinal monitoring strategies (189, 190). In parallel, data-driven approaches may assist in optimizing manufacturing workflows and reducing batch-to-batch variability, although their clinical utility remains to be fully established.

Finally, ethical, safety, and regulatory considerations remain central as Treg therapies become increasingly complex. Strategies involving extensive genetic modification or stem cell-derived platforms necessitate careful assessment of genomic integrity, long-term safety, and quality control. Addressing these challenges through conservative clinical trial design, robust manufacturing standards, and extended follow-up will be essential to ensure responsible and reproducible translation of regulatory T cell therapies.
